# Spatial Lipidomics Reveals Lipid Changes in the Cotyledon and Plumule of Mung Bean Seeds during Germination

**DOI:** 10.1021/acs.jafc.3c06029

**Published:** 2023-11-29

**Authors:** Peisi Xie, Jing Chen, Pengfei Wu, Zongwei Cai

**Affiliations:** † Ministry of Education Key Laboratory of Analytical Science for Food Safety and Biology, Fujian Provincial Key Laboratory of Analysis and Detection Technology for Food Safety, College of Chemistry, Fuzhou University, Fuzhou, Fujian 350116, China; ‡ State Key Laboratory of Environmental and Biological Analysis, Department of Chemistry, Hong Kong Baptist University, Hong Kong, Special Administrative Region 999077, China; § College of Forestry, Nanjing Forestry University, Nanjing, Jiangsu 210018, China

**Keywords:** lipidomics, mass spectrometry imaging, cotyledon and plumule, mung bean seeds, germination

## Abstract

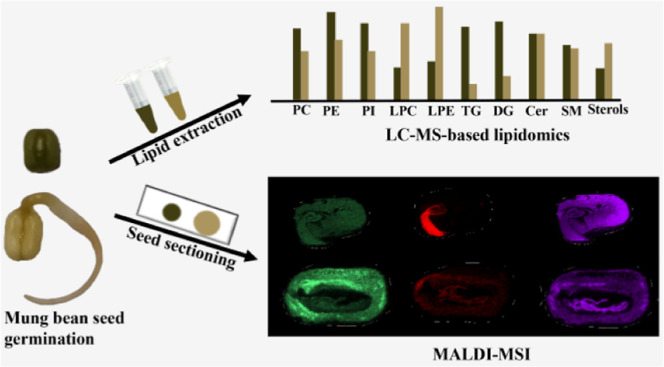

Seed germination is a vital process in plant development involving dynamic biochemical transformations such as lipid metabolism. However, the spatial distribution and dynamic changes of lipids in different seed compartments during germination are poorly understood. In this study, we employed liquid chromatography/mass spectrometry (LC/MS)-based lipidomics and MALDI mass spectrometry imaging (MSI) to investigate lipid changes occurring in the cotyledon and plumule of mung bean seeds during germination. Lipidomic data revealed that the germination process reduced the levels of many glycerolipids (e.g., triglyceride) and phosphatidylglycerols (e.g., phosphatidylcholine) while increased the levels of lysophospholipids (e.g., lysophosphatidylcholine) in both the cotyledon and plumule. Sphingolipids (e.g., sphingomyelin) displayed altered levels solely in the plumule. Sterol levels increased in the cotyledon but decreased in the plumule. Further imaging results revealed that MALDI–MSI could serve as a supplement and validate LC–MS data. These findings enhance our understanding of the metabolic processes underlying seedling development, with potential implications for crop improvement and seed quality control.

## Introduction

Mung bean seeds are widely consumed due to their nutritional value and health benefits. They are rich in proteins, carbohydrates, vitamins, and minerals, making them an important component of human diets in many cultures.^1^ In addition to their dietary significance, mung bean seeds are also utilized for various purposes, including sprouting for culinary uses and as a source of raw material in the food and beverage industries.^2^ The internal structure of mung bean seeds consists of a protective seed coat, embryo, and cotyledon.^3^ The seed coat shields the inner parts from external damage. The embryo contains the future plant, including the radicle, hypocotyl, and plumule. The cotyledon stores nutrients such as carbohydrates, proteins, and fats, providing nourishment for germination. Seed germination is the process that begins with the absorption of water by the seed and concludes with the radicle (embryonic root) breaking through the seed coat.^4^ Understanding the physiological and biochemical changes that occur during seed germination is crucial to optimizing their utilization and improving crop production.

Lipids play a crucial role in energy storage, membrane synthesis, and signal transduction during seed germination.^5−7^ When mung bean seed absorbs water, it triggers the activation of lipases, which are responsible for breaking down stored lipids. These lipids, mainly in the form of triglycerides (TGs), are stored in the cotyledons of seeds.^8^ As the lipases break down the TGs, they release free fatty acids and glycerol. Free fatty acids are transported to the mitochondria to generate acetyl coenzyme A (acetyl-CoA) and produce ATP to provide energy for the growth of seedlings. This breakdown of fatty acids continues until the stored lipids are sufficiently depleted. Additionally, during germination, seeds synthesize new lipids to support their growth.^9−11^ Acetyl-CoA is used in the biosynthesis of fatty acids and phospholipids. These lipids are essential for the formation of cell membranes and various cellular functions. Besides, one previous work revealed that during the germination of whole mung seeds, there was a reduction in the levels of phospholipids and TGs, while there was an increase in monoglycerides (MGs) and sterols.^12^ However, to the best of our knowledge, no study has investigated the dynamic changes of lipids in different seed compartments during the germination of mung bean seeds.

Matrix-assisted laser desorption/ionization mass spectrometry imaging (MALDI–MSI) is a technique used to analyze and visualize the spatial distribution of biomolecules in different biological samples.^13−16^ It allows for simultaneous measurement of mass and spatial information on molecules, making it a powerful tool in various fields of research.^17−19^ Increasing numbers of studies have performed MSI analysis to investigate the connections among endogenous molecules within seeds and the regulation of metabolic processes (e.g., germination) in seed cells. The initial MSI studies in sections of embryos of cotton seeds revealed that the cotyledons and embryonic axis of the cotton embryo displayed distinct enrichments of monounsaturated, polysaturated, cyclic, and saturated lipids, indicating an uneven distribution of metabolic processes for these metabolites within the embryo tissue.^20^ Bhandari et al. investigated the metabolic changes in oilseed rape during germination.^21^ They found that in the mature seed, some metabolites (e.g., the spermidine conjugate) were mainly distributed in the hypocotyl-radicle area. However, these metabolites were found to be located in the emerging radicle of germinating seeds. Gupta et al. applied MALDI–MSI to determine the changes of lipids in two types of barley seeds with contrasting germination characteristics.^22^ More than 200 lipid species had significant alternations in spatial distributions between the different seeds. However, no studies have applied MSI to investigate the lipid distribution or variations of lipids in mung seeds during germination.

In this study, we aim to examine the changes in lipid metabolism and distributions in the cotyledon and plumule of mung bean seeds during germination. Liquid chromatography–tandem mass spectrometry (LC–MS)-based lipidomics and MALDI–MSI were applied to investigate the variations of different lipid classes and changes in spatial distributions of different lipid species between two regions of mung bean seeds in two different germinating days. Segmentation analysis was performed to distinguish different regions in the mung bean seeds. The detected number of lipid species between LC–MS and MALDI–MS analyses was also compared. This work may contribute to the advancement of seed germination research and enhance our understanding of the biochemical processes underlying plant development.

## Experimental Section

### Chemicals

Isopropanol, methanol, ammonium acetate, chloroform, acetonitrile, methanol, formic acid, and trifluoroacetic acid were obtained from Merck (Darmstadt, Germany). 1,5-Diaminonaphthalene (1,5-DAN) and 2,5-dihydroxybenzoic acid (DHB) were purchased from Sigma-Aldrich (St. Louis, U.S.A.). TG(15:0/15:0/15:0), phosphatidylcholine(19:0/19:0) [PC(19:0/19:0)], and ceramide(d18:1/17:0) [Cer(d18:1/17:0)] were obtained from Avanti Polar Lipids (Alabaster, U.S.A.).

### Seed Germination

Mung bean seeds (Hart Limited, China) were purchased from a local supermarket in Hong Kong. Seeds were washed with deionized water three times and cultured in 24-well plates. On day 0, a total of 1 mL of deionized water was added into each well containing one seed. Plates were wrapped with aluminum foil to avoid light exposure and placed into a plant incubator at 25 °C. Deionized water was replaced every 24 h. On day 3, the aluminum foils were removed, and seeds were grown in the incubator with a 12 h dark/light cycle.

### Lipid Extraction

At 24 and 96 h in culture, cotyledons and plumules in mung bean seeds were carefully dissected and collected. Due to the lightweight nature of the plumule samples of mung beans, we combined eight individual samples into one composite sample. Each group contained six composite samples. Each composite sample was crushed, mixed, and weighed (about 10 mg in wet weight). Lipid extraction was carried out according to a method reported in our previous work.^23^ Briefly, for each sample in a 2 mL tube, a total of 750 μL of 80% cooled aqueous methanol was added. These samples were crushed using a tissue disruptor and repeatedly freeze–thawed five times using liquid nitrogen. Tubes were centrifuged (13,500 rpm, 4 °C, 15 min), and the suspension was collected into a new tube. Chloroform (450 μL) was added and mixed well with the suspension, followed by the addition of deionized water (150 μL). The tube was vortexed for 2 min, left at 25 °C for 6 min, and centrifuged (13,500 rpm, 4 °C, 15 min). Lipids in the bottom layer and proteins in the middle layer were collected. Bicinchoninic acid assay was carried out to determine the protein concentration in each sample. Lipid liquids were spun dried in a freeze-dryer and dissolved in 150 μL of solvent containing 65% acetonitrile, 30% isopropanol, 5% deionized water, 2 μg/mL of TG(15:0/15:0/15:0), Cer(d18:1/17:0), and PC(19:0/19:0). The mixture is sonicated until the yellow lipid precipitate is completely dissolved and further vortexed and centrifuged (13,500 rpm, 4 °C, 15 min). A total of 100 μL of supernatant was used for further LC–MS/MS analysis.

### LC–MS/MS and Data Analyses

The lipid analysis was performed using an UPLC system connected with a Q Exactive Focus mass spectrometer (Thermo Scientific, U.S.A.). Lipids were separated by using a C18 column (Waters, Germany). All of the experiments were carried out in the Institute of State Key Laboratory of Environmental and Biological Analysis in Hong Kong Baptist University. The mobile phase A consisted of a mixture of isopropanol/acetonitrile (90:10, v/v) containing 0.1% formic acid and 10 mM ammonium formate. The mobile phase B was composed of a mixture of acetonitrile/water (60:40, v/v) containing 0.1% formic acid and 10 mM ammonium formate. The flow rate was set at 0.26 mL/min with an injection volume of 10 μL. The gradient elution conditions were as follows: 0–1 min (30% A), 1–2 min (30 to 45% A), 2–7 min (45 to 70% A), 7–9 min (70 to 85% A), 9–17 min (85 to 100% A), 17–19 min (100% A), 19–20 min (100 to 30% A), and 20–24 min (30% A). The temperature of the sampler and column were set at 8 and 50 °C, respectively. The initial stage of the running sequence included two blank samples and five quality control (QC) samples. Additionally, one QC sample was arranged into six samples.

LipidSearch software was employed for ion peak extraction and alignment of lipid profiling data. TG(15:0/15:0/15:0), Cer(d18:1/17:0), and PC(19:0/19:0) were used as internal standards for glycerolipids (GLs), sphingolipids (SPs), and glycerophospholipids (GPs), respectively. The extracted peak areas of different lipid species in different parallel samples were calibrated by their protein contents. Partial least-squares discriminant analysis and pathway analysis were carried out by MetaboAnalyst 5.0 and LIPID MAPS. Significantly changed lipid species and classes were identified as the threshold of fold change (FC, FC > 1.5 or <0.67) and *p* value (*p* < 0.01). Identification of these lipid species involved a manual verification process, wherein the MS and MS/MS information from the raw data was compared with the LipidSearch database. The final data were reported as the mean ± standard error of the mean (SEM).

### Sample Preparation for MALDI–MSI Analysis

At 24 and 96 h in culture, mung bean seeds were collected, washed with deionized water three times, and split in half evenly. The preparation of frozen sections of seeds was carried out according to a method reported in our previous work with a minor modification.^24^ Briefly, a total of 800 μL of 2% aqueous carboxymethyl cellulose was added onto the cryostat support, and the liquid was allowed to cool until it formed ice. A mold was prepared by slicing this ice block and marked with an oil pen. This mold was taken out from the support holder, and half of the mung bean seed was placed onto the prepared frozen mode and fixed by adding 20 μL of deionized water to the bottom of the seed. This mold was reinserted into the support holder based on the former label. The seeds were sliced into sections with a thickness of 14 μm and dried in a vacuum dryer for 30 min.

The DHB (15 mg/mL) matrix used in positive ionization mode was dissolved in methanol/H_2_O (70:30, v/v) containing 0.1% trifluoroacetic acid. 1,5-DAN matrix used in negative ionization mode was prepared at a concentration of 5 mg/mL in methanol/H_2_O (70:30, v/v). These matrices were sprayed onto seed sections by using an instrument named HTX H5 sprayer. A total of 18 and 15 spray cycles were applied for DHB and 1,5-DAN matrices, respectively. Other main spraying parameters including nozzle temperature (66 °C), nozzle height (4 cm), flow rate (0.03 mL/min), track spacing (2 mm), air pressure (10 psi), moving velocity (2000 mm/min), and dry time (20 s) were used in this experiment.

### Data Acquisition and Processing for MALDI–MSI

The acquisition of MSI data of seed sections was performed on a timsTOF flex MALDI-2 instrument operating at 10 kHz in both positive and negative ionization modes, covering a mass range of *m*/*z* 100–1050. All data were acquired by using a raster size of 50 × 50 μm in M5 small mode with a 90% laser power. The instrument was calibrated by the Agilent calibration mix and operated using the following device settings: collision radio frequency (RF), 900 voltage peak–peak (Vpp); funnel 1 RF, 180 Vpp; multipole RF, 1200 Vpp; transfer time, 110 μs; funnel 2 RF, 180 Vpp; prepulse storage, 11 μs; and laser shots, 350.

The raw MSI data were imported into the software of SCiLS Lab MVS 2023b premium three-dimensional and normalized to the method of the root-mean-square. The potential lipid list was created by manually annotating *m*/*z* peaks that exhibited spatial localization across the seed sections. The list underwent additional analysis to assign ion adducts ([M + Na]^+^, [M + K]^+^, and [M + H]^+^ for positive ionization mode and [M – H]^−^ for negative ionization mode) by searching one online database (LIPMAPS) with a mass-to-charge ratio deviation less than 5 ppm. Spatial segmentation of seed sections was carried out under the method of segmentation analysis with bisecting *k*-means and weak denoise. The probabilistic latent semantic analysis (pLSA) was performed to investigate the differences in lipid metabolism in cotyledons and plumules of seed sections between germinating days 1 and 4. Each group contained six replicates. All significantly changed lipid species were identified as having a threshold of FC > 1.5 or <0.67 and a *p* value (*p* < 0.01).

## Results and Discussion

### Seed Growth during Germination

**Figure 1:**
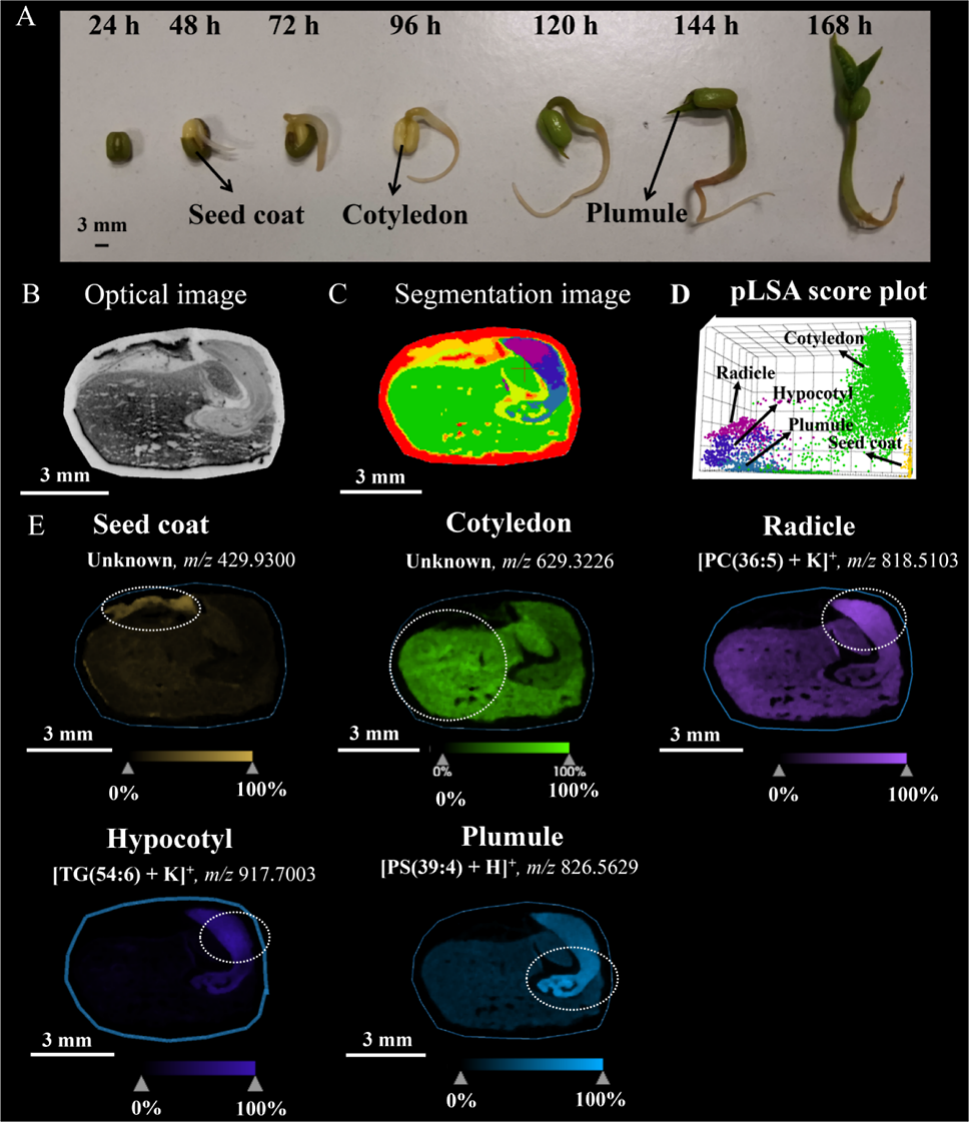
(A) Physical pictures of mung bean seeds on different germinating days. Optical image (B), segmentation image (C), and pLSA score plot (D) of the section of the mung bean seed on germinating day 1. (E) Representative structure-specific ion images in the mung bean seed on germinating day 1. Ions at *m*/*z* 429.9300, 629.3226, 818.5103, 917.7003, and 826.5619 were mainly distributed in the seed coat, cotyledon, radicle, hypocotyl, and plumule, respectively. Scale bar = 3 mm for all seed sections.

Physical alterations related to germination were observed during different germinating times. As shown in Figure 1A, from 24 to 96 h, the mung bean seed absorbed water and swelled, resulting in an increase in size. The outer seed coat cracked, and the radicle and hypocotyl emerged from the seed. From 96 to 144 h, due to the shedding of the seed coat, the cotyledon became visible. The radicle and hypocotyl exhibited continuous growth, progressively forming more mature structures of the roots and shoot. Besides, the plumule broke through the cotyledon and gradually grew upward. All the internal structures except the radicle turned green and started to photosynthesize and provided energy for further growth.^25^ At 168 h, the seedling continued to grow, with the plumule expanding and developing more complex structures. The radicle became more extensive, allowing for better nutrient absorption.^26^

### Segmentation Analysis of Seed Sections

The identification of several internal structures of mung beans was also proven by the segmentation analysis of frozen seed sections of mung beans at germinating day 1. As shown in Figure 1B,C, five internal structures including the seed coat (yellow), cotyledon (green), radicle (magenta), hypocotyl (purple), and plumule (blue) were found in seed sections. The result of the pLSA score plot (Figure 1D) demonstrated clear distinctions among five internal structures, suggesting significant variations in the lipid metabolism of cells within each structure. Based on the results of spatial segmentation analysis, these five structures were selected for further analysis of the loading plot to identify ions that played major roles in distinguishing these structures. These ions were outside the 95% confidence ellipse (Figure S1). For instance, in positive ionization mode (Figure 1E), the unknown compound ion at *m*/*z* 429.9300 was found to mainly locate in the seed coat; the unknown ion at *m*/*z* 629.3226 was observed to predominantly distribute in the cotyledon; the ion at *m*/*z* 818.5103 was assigned to [PC(36:5) + K]^+^ and found to be intense in the radicle; the ion at *m*/*z* 917.7003 was assigned to [TG(54:6) + K]^+^ and observed to mainly distribute in the hypocotyl, and the ion at *m*/*z* 826.5629 was assigned to [PS(39:4) + H]^+^ and observed to largely locate in the plumule.

### Partial Least-Squares Discriminant Analysis of the Cotyledon and Plumule in Seeds between Germinating Days 1 and 4

During the germination of mung bean seeds, different structures inside the seeds serve various functions.^27,28^ For instance, cotyledons are the primary storage organs within the seed. They contain the necessary nutrients, such as carbohydrates, proteins, and lipids, which provide energy for the developing embryo during germination. The plumule is the embryonic shoot located between the cotyledons.^29^ It consists of the epicotyl and first true leaves. The germination process involves dynamic changes in lipid metabolism, which plays a crucial role in seedling growth.^30^ In order to investigate the lipid changes in different structures in mung bean seeds during germination, we performed LC–MS/MS-based lipidomic analysis between cotyledons and plumules in seeds for between 24 and 96 h. Samples at these two time points were selected for two reasons. First, samples at 24 h were chosen to capture early lipid composition during the germination process, as the seeds begin to metabolize stored lipids for energy. Samples at 96 h were selected to capture more pronounced lipid changes that occur during later stages of germination. By this time, the seeds had undergone significant metabolic activity, and the lipid profile was expected to exhibit more noticeable alterations. Second, selecting seeds at 24 and 96 h is more advantageous for preparing frozen sections for the MSI study. Seeds at 0 h have a low moisture content, making it difficult to prepare thin frozen sections. Seeds at 120 h, on the other hand, have separated plumules and cotyledons (Figure 1A), which makes it challenging to prepare frozen sections containing both tissues.

**Figure 2:**
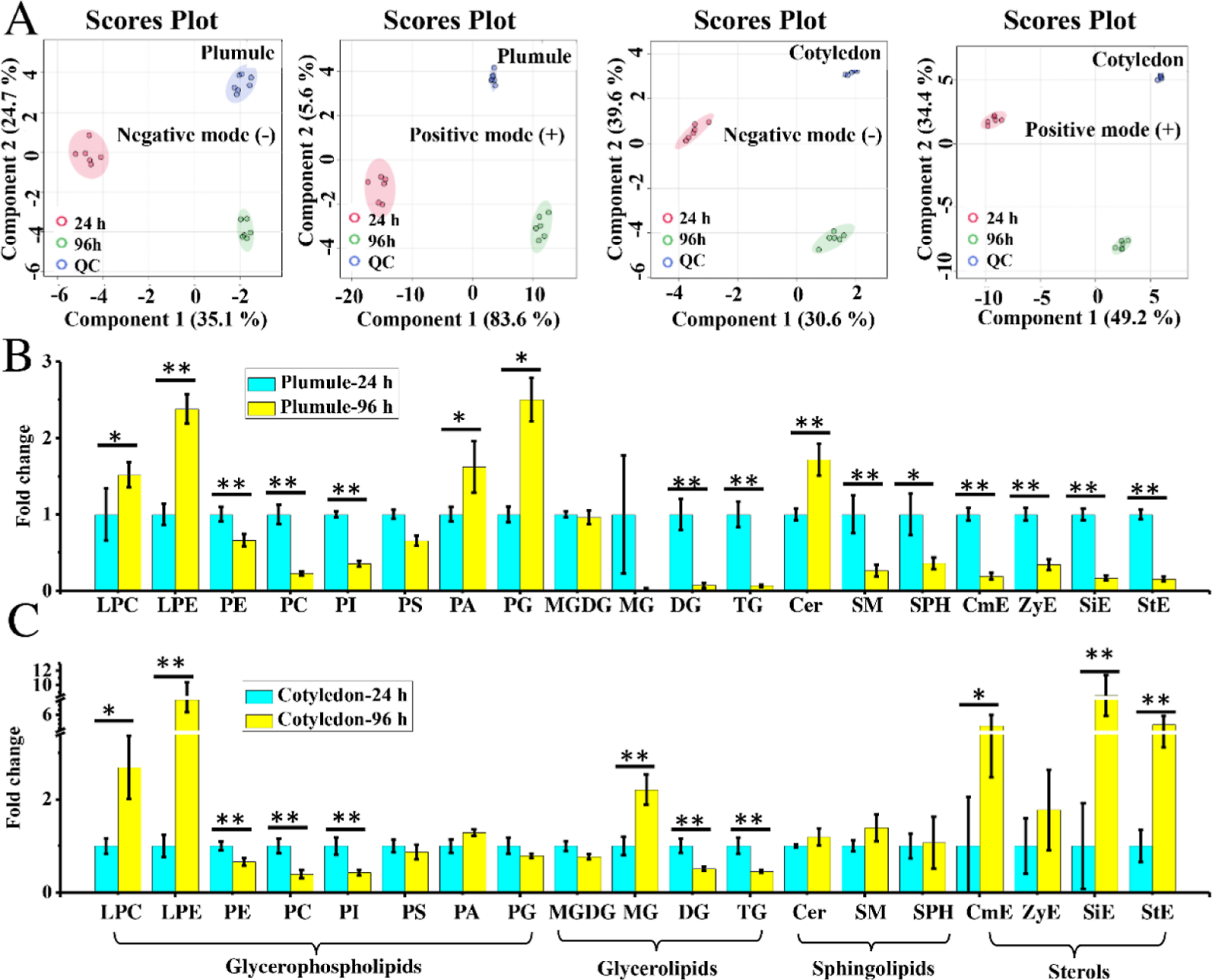
(A) Partial least-squares discriminant analysis of score plots based on LC–MS-based lipidomic data of the plumule and cotyledon in mung bean seeds between germination for 24 and 96 h in negative and positive ionization modes. Fold changes of many lipid classes belonging to GPs, GLs, SPs, and sterols in the plumule (B) and cotyledon (C) in mung bean seeds between germinating 24 and 96 h. Each group contained six replicates. (*p < 0.01 and **p < 0.001.)

As shown in Figure S2, the results of cross-validation of partial least-squares discriminant analysis (*Q*^2^ and *R*^2^*Y* > 0.8) demonstrated the satisfactory predictive capability for lipid profiling of cotyledons and plumules in both negative and positive ionization modes. The QC samples that were clustered (Figure 2A) provided evidence of a consistent instrumental performance. Clear separations were observed in plumules and cotyledons of seeds between different germinating timings (Figure 2A), suggesting that germinating processes caused obvious disturbances in the lipid profiles of both structures.

### Germinating Process Altered Levels of Various Lipid Classes in the Cotyledon and Plumule of Mung Bean Seeds

In the lipid analysis, 586 lipids containing 222 GPs, 61 SPs, 290 GLs, and 13 sterols were detected in the plumule. In the cotyledon, 502 lipids including 216 GPs, 53 SPs, 226 GLs, and 7 sterols were detected. A total of 473 (Table S1) and 353 (Table S2) lipids were identified as significantly changed based on the criteria of FC < 0.67 or >1.5 and *p* < 0.01 in the plumule and cotyledon, respectively. Among these lipids (Table S1), 34 were SPs in the plumule, consisting of 32 Cers, 1 sphingomyelin (SM), and 1 sphingosine (SPH); 161 lipids belonged to GPs in the plumule, including 13 lysophosphatidylcholines (LPCs), 7 lysophosphatidylethanolamines (LPEs), 13 phosphatidic acids (PAs), 45 PCs, 34 phosphatidylethanolamines (PEs), 16 phosphatidylglycerols (PGs), 26 phosphatidylinositols (PIs), and 7 phosphatidylserines (PSs); 265 lipids were GLs in the plumule, including 74 diacylglycerols (DGs), 1 MG, 13 monogalactosyldiacylglycerol (MGDG) and 177 TGs; and 13 lipids belonged to sterols in the plumule, consisting of 4 campesterol ester (CmE), 4 zymosteryl ester (ZyE), 3 sitosteryl ester (SiE), and 2 stigmasteryl ester (StE). For 348 lipids in cotyledon (Table S2), 30 lipids including 27 Cers, 2 SPHs, and 1 SM were SPs; 168 lipids including 11 LPCs, 8 LPEs, 14 PAs, 60 PCs, 29 PEs, 12 PGs, 27 PIs, and 7 PSs were GPs; 150 lipids containing 34 DGs, 1 MG, 6 MGDG, and 109 TGs belonged to GLs; and the remaining 5 lipids including 1 CmE, 2 SiE, and 2 StE were sterols.

Previous works showed that different lipid classes have different biological functions in plant seeds.^13,14^ Hence, we analyzed the levels of various lipid classes in plumule and cotyledon in mung bean seeds during germinating days 1 and 4. As shown in Figure 2B,C, for GPs, elevated levels of LPC and LPE and reduced levels of PC, PE, and PI were both observed in the plumule and cotyledon, while significantly increased levels of PA and PG were only found in the plumule. For GLs, reduced levels of DG and TG were both found in the plumule and cotyledon, while an increased level of MG was observed only in the cotyledon. For SPs, no significant changes in levels of all three lipid classes (Cer, SM, and SPH) were found in the cotyledon, while an increased level of Cer and reduced levels of SM and SPH were observed in the plumule. For sterols, interestingly, reduced levels of all four lipid classes including CmE, ZyE, SiE, and StE were found in the plumule, while increased levels of three lipid classes containing CmE, SiE, and StE were observed in the cotyledon.

### Lipid Pathways of the Plumule and Cotyledon Involved in Mung Bean Seed Germination

**Figure 3:**
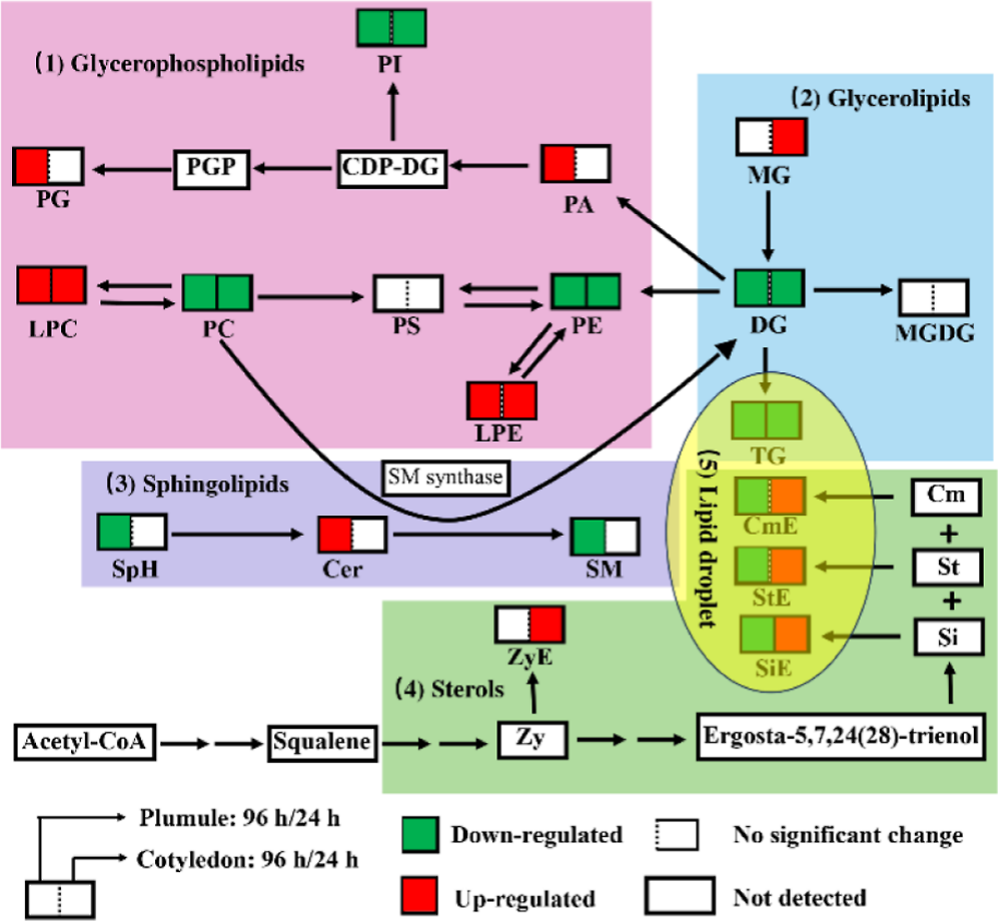
Lipidomic networks of the plumule and cotyledon in mung bean seeds during germination. The reduced and increased levels of lipid classes are represented by the green and red boxes, respectively.

The changes in lipid classes observed in the cotyledon of our study were found to be quite similar to those reported in a previous study on whole mung beans.^12^ This similarity may be attributed to the fact that the cotyledons of mung bean seeds occupy approximately 70% of the total volume. One other study reported that in the whole borage seed, the germinating process led to the downregulated levels of TG, PC, PS, PE and the upregulated levels of PA, while the level of DG remained unchanged.^31^ These results were somewhat inconsistent with our results, which may be due to the difference in growth and development between different plant species. As shown in Figure 3, the reduced levels of DG and TG in both the cotyledon and plumule during germination can be explained by the mobilization and utilization of these lipids as an energy source for seed germination and early seedling growth. During seed germination, stored lipids in both the cotyledon and plumule are mobilized to provide energy for metabolic processes required for seedling establishment.^30,32^ DG and TG are major storage lipids in seeds, and their breakdown releases fatty acids that can be used for energy production through β-oxidation.^32^ In GPs (Figure 3), the downregulated levels of PC, PE, and PI and upregulated levels of LPE and LPC in cotyledon and plumule suggested the remodeling of the lipid composition to meet the demands of seedling growth and development. PC, PE, and PI are essential structural components of cell membranes.^33^ As the seed grows, it requires the formation of new membranes for the expansion of cells and the establishment of new cellular structures.^34^ The breakdown of PC, PE, and PI may provide raw materials, such as fatty acids, for the synthesis of new membranes. Lysophospholipids (e.*g.*, LPC and LPE) are intermediates in the degradation of phospholipids.^35^ They are formed when one fatty acid is removed from the phospholipid molecule. The breakdown of phospholipids may lead to elevated levels of lysophospholipids (Figure 3).

In this study, elevated levels of PA and PG were observed in the plumule but not in the cotyledon (Figure 3). This may be because PA and PG are known to play regulatory roles in plant growth and development.^36^ PA is involved in signaling pathways related to stress responses and growth promotion, while PG is a precursor for the synthesis of important lipids, such as chloroplast lipids.^36,37^ The plumule, being the embryonic shoot, is responsible for initiating and supporting the growth of the seedling. It requires higher levels of energy and membrane lipids for cell division, elongation, and differentiation. This increased metabolic activity might lead to higher synthesis and accumulation of PA and PG in the plumule compared to the cotyledon. Besides, for SPs, the germination process altered levels of Cer, SM, and SPH in the plumule (Figure 3), suggesting that the cotyledon may have a more stable lipid composition during germination, while the plumule undergoes specific lipid changes to support its growth and development. Cer is a component of cellular membranes and is involved in various cellular processes, including cell signaling and apoptosis.^38^ The increased levels of ceramide in the pluma indicated a heightened need for membrane remodeling and cell signaling during germination. SM and SPH are known to play important roles in membrane structure and signaling.^39^ The reduced levels of SM and SPH in the plumule suggested a decreased requirement for membrane stability and signaling in this tissue during germination.

Sterols can be synthesized by several complex biochemical pathways (Figure 3). Generally, acetyl-CoA can be converted into squalene, which is a precursor in the synthesis of phytosterols.^40^ Squalene undergoes various modifications, such as oxidation and reduction, to produce different sterols [e.g., zymosteryl (Zy), campesterol (*Cm*), sitosteryl (Si), and stigmasteryl (St)].^41^ These sterols can be further converted into their ester forms (e.g., ZyE, CmE, SiE, and StE) by bonding to different fatty acids.^42^ Sterols are primarily found in plant cell membranes and play essential roles in regulating the fluidity and stability of membranes.^42^ The decreased levels of ZyE, CmE, SiE, and StE may indicate that the membranes of the plumule are becoming more fluid and dynamic, allowing for the growth and expansion of cells. The cotyledon is responsible for providing nutrients to support the developing seedling. The increased levels of CmE, SiE, and StE in the cotyledons suggest an active metabolic state, possibly related to nutrient storage and transport. Sterols may play a role in maintaining membrane integrity and regulating the nutrient uptake and transport within mung bean seeds.

### Comparison of Lipids Detected by MALDI–MS and LC–MS in Mung Bean Seeds

**Figure 4:**
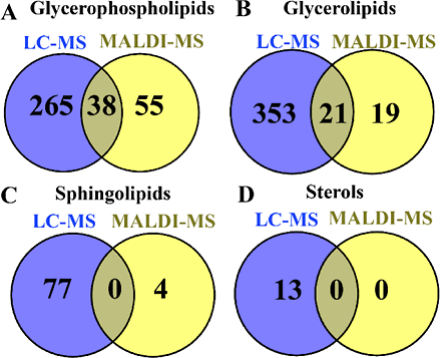
Venn diagrams for four lipid categories, including (A) GPs, (B) GLs, (C) SPs, and (D) sterols. The diagrams show the number of shared lipid species in different categories between LC–MS and MALDI–MS.

It is worth noting that while LC–MS with an electrospray ionization source is known for its high sensitivity and ability to identify polar and semipolar compounds, MALDI–MSI with a high mass resolution offers excellent sensitivity and spatial resolution for the analysis of both polar and nonpolar species.^43−45^ Hence, we suspected that MALDI–MS combined with LC–MS could broaden the detection range of lipids in the mung seeds. To confirm this, we first compared the detected lipid numbers and classes in mung bean seeds between MALDI–MS and LC–MS. As shown in Figure 4A–C, a total of 93, 40, and 4 lipid species belonging to GPs, GLs, and SPs were detected by MALDI–MS. The lipid species in GPs, GLs, and SPs detected by LC–MS were 303, 374, and 77, respectively. The numbers of shared lipid species in GPs, GLs, and SPs between MALDI–MS and LC–MS were 38, 21, and 0, respectively. In GPs, the 38 shared lipid species included 3 LPEs, 2 LPCs, 11 PIs, 9 PEs, 13 PCs, 8 Pas, and 3 PGs (Figure S3). Three lipid classes including lysophosphatidic acid (LPA), lysophosphatidylglycerol (LPG), and lysophosphatidylinositol (LPI) were only detected in MALDI–MS (Figure S3). In sterols, all lipid species including 4 CmE, 4 ZyE, 3 SiE, and 2 StE were only detected in LC–MS (Figure 4D).

In SPs, no lipid species were shared between LC–MS and MALDI–MSI (Figure 4C). Two lipid classes, including Cer and SPH, were solely detected by LC–MS (Figure S3). In GLs, 21 lipid species, including 1 DG and 20 TGs, were shared between the two methods (Figures 4B and S3). Two lipid classes including MG and MGDG were detected only by LC–MS (Figure S3). Taken together, our results suggest that both MALDI–MS and LC–MS are capable of detecting many lipid species belonging to different lipid classes in mung bean seeds but each with its own unique detected compounds. The combination of both methods can cover a wider range of lipids in mung bean seeds, which may allow us to comprehensively understand the dynamic changes in lipid composition and distribution during seed germination.

### MALDI–MSI Analysis of the Cotyledon and Plumule in Seeds between Germinating Days 1 and 4

**Figure 5:**
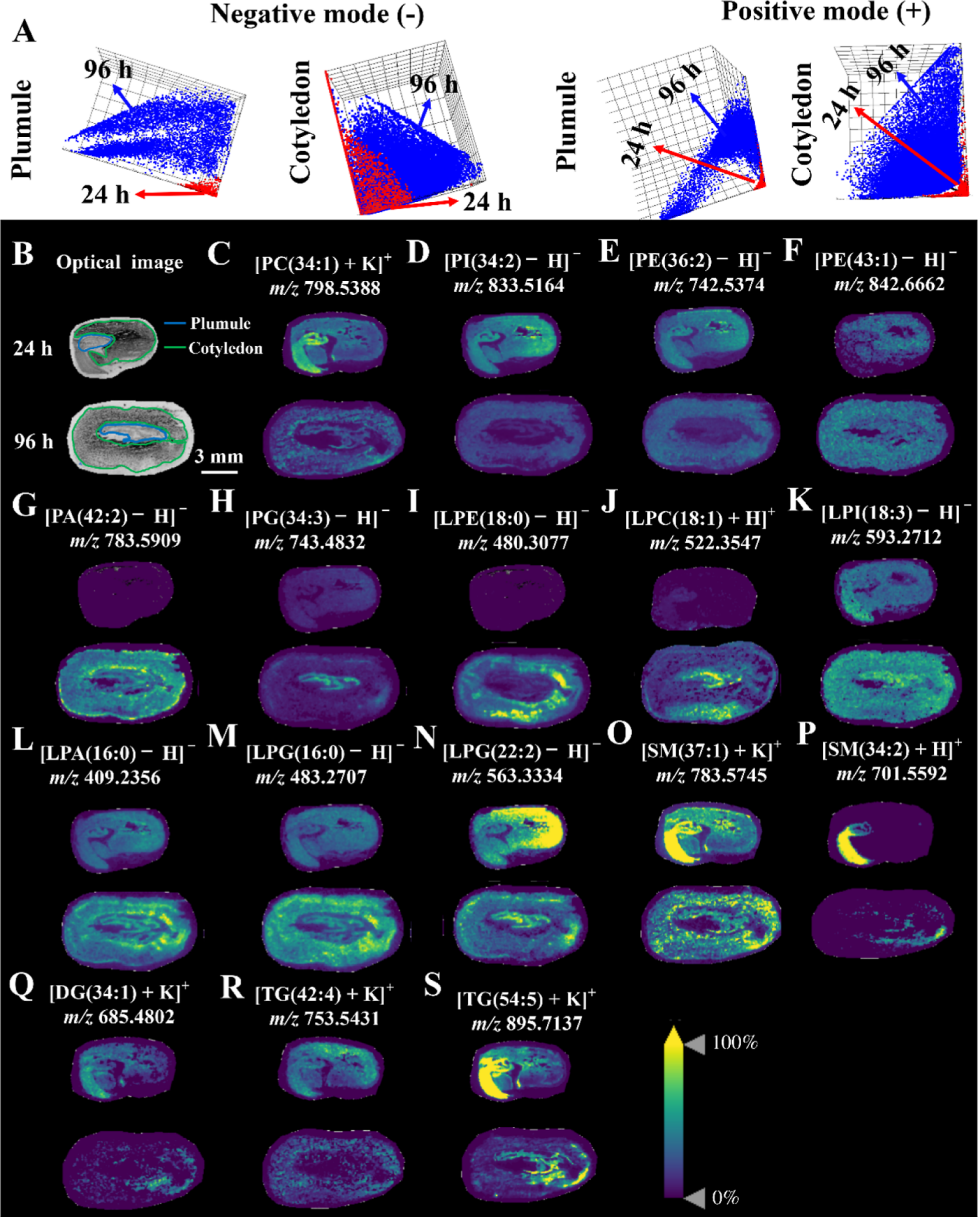
(A) pLSA score plots based on MALDI–MSI data of the plumule and cotyledon in mung bean seeds between 24 and 96 h of germination in negative and positive ionization modes. Optical image (B) and representative images (C–S) of different lipid ion species in mung bean seeds between 24 and 96 h of germination. Scale bar = 3 mm for all seed sections.

The results of MS-based lipidomics showed that the levels of various lipid classes in the cotyledon and plumule in mung bean seeds were altered during germination (Figure 2B,C). To study the changes of abundance and spatial distribution of many species in these lipid classes in the two structures, we conducted MALDI–MSI analysis of seed sections between germinating days 1 and 4. Two common organic matrices (DHB for positive ionization mode and 1,5-DAN for negative ionization mode) were utilized in this study. The results of pLSA score plots in MALDI–MSI data showed obvious separations between the plumule and cotyledon in different germinating timings in two ionization modes (Figure 5A), suggesting alterations in the lipid metabolism in the two structures during germination. Similar to the results in the LC–MS data (Figure 2C), during germination, intensities of all 11 significantly altered PCs [e.g., PC(34:1)] were downregulated in the cotyledon of mung bean seeds (Figure 5C and Table S3). In the plumule, the intensities of seven PCs [e.g., PC(36:4)] were downregulated, while the signal intensity of one PC (40:2) was upregulated (Figure S4A,B and Table S4). A total of 10 PIs [e.g., PI (34:2)] had reduced intensities in both the plumule and cotyledon (Figure 5D and Tables S3 and S4). For PEs, 5 lipid species [e.g., PE (36:2)] had reduced intensities in two structures, while 1 lipid species [e.g., PE (43:1)] showed increased intensities in the plumule but not in the cotyledon (Figure 5E,F, Table S3 and S4). A total of 5 and 2 lipid species ((e.g., PA(42:2)) showed increased intensities in the plumule and cotyledon, respectively (Figure 5G, Table S3 and S4). For PGs, two lipid species, (PG(34:3) and PG(32:0), had increased signal intensities in the plumule (Figure 5H, Figure S4C, and Table S4). The signal intensities of one species [PG(32:0)] and one species [PG(34:2)] in the cotyledon were increased and decreased, respectively (Figure S4C,D and Table S3).

For lysophospholipids, increased intensities of 3 LPEs [e.g., LPE(18:0)] were found in both the plumule and cotyledon (Figure 5I and Tables S3 and S4). A total of 5 and 4 LPCs [e.g., LPC(18:1)] had increased signal intensities in the cotyledon and plumule, respectively (Figure 5J and Tables S3 and S4). Other lysophospholipids including LPI, LPA, and LPG were detected in MALDI–MSI but not in LC–MS (Figure S3). For LPI, intensities of all 6 and 4 significantly altered lipid species [e.g., LPI(18:3)] were downregulated in the cotyledon and plumule, respectively (Figure 5K and Tables S3 and S4). These may be due to the degradation of PI in two structures (Figure 3B,C). For LPA, two detected species [LPA(16:0) and LPA(18:2)] all had increased signal intensities in two structures (Figures 5L and S4E and Tables S4 and S3). For LPG, two significantly altered species [LPG(16:0) and LPG(22:2)] had increased intensities in the plumule (Figure 5M,N and Table S4). In the cotyledon, the signal intensity of one species [LPG(16:0)] increased, while the signal intensity of another species [(LPG(22:2)] reduced (Figure 5M,N and Table S3). The increased intensities of LPA and LPG in the plumule suggested an enhanced synthesis of PA and PG in this structure (Figure 3).

For SPs, a total of 2 lipid species, SM(37:1) and SM(34:2), had reduced intensities in the plumule, while one lipid species, SM(34:2), had increased intensity in the cotyledon (Figure 5O,P and Tables S3 and S4). For GLs, intensities of 2 and 3 lipid species [e.g., DG(34:1)] in DG in the plumule and cotyledon were downregulated (Figure 5Q and Tables S3 and S4). In the plumule, a total of 8 TGs [e.g., TG(42:4)] had reduced intensities (Figure 5R and Table S4), while one species [TG(54:8)] had increased intensity (Figure S4F and Table S4). In the cotyledon, intensities of 21 TGs [e.g., TG(54:5)] were reduced (Figure S5 and Table S3), while intensities of 2 TGs [TG(40:1) and TG(41:2)] were increased (Figure S4G,H and Table S3). Taken together, the above results showed that MSI data could act as a validation and supplement to the LC–MS-based lipidomic data.

This study utilized LC/MS-based lipidomics and MALDI MSI to investigate the spatial distribution and dynamic changes of lipids in the cotyledon and plumule of mung bean seeds during germination. The results revealed that the germination process led to the downregulation of many GLs (e.g., TG) and GPs (e.g., PC) and the upregulation of lysophospholipids (e.g., LPC) in both compartments. Additionally, some lipid classes (e.g., PG, SM, and Cer) displayed altered levels solely in the plumule. Sterol levels increased in the cotyledon but decreased in the plumule. Further MALDI–MSI analysis demonstrated its ability to improve the accuracy and broaden the detection range of lipidomic analysis. These findings provide valuable insights into the metabolic processes underlying seedling development, which may contribute to crop improvement and seed QC.

## Supporting Information Available

Loading plot of five internal structures in mung bean seeds; cross-validations of partial least-squares discriminant analysis of score plots; Venn diagrams for various lipid classes; representative images of different lipid ion species in mung bean seeds; information of lipid markers in the plumule and cotyledon between 24 and 96 h identified by UPLC–MS/MS; and information of significantly changed lipids in the cotyledon and plumule of mung bean seeds between germination days 1 and 4 identified by MALDI–MSI (PDF)

## Abbreviations

TGs: triglycerides
MGs: monoglycerides
MALDI–MSI: matrix-assisted laser desorption/ionization mass spectrometry imaging
LC–MS: liquid chromatography–mass spectrometry
MALDI–MSI: matrix-assisted laser desorption ionization–mass spectrometry imaging
1: 5-DAN, 1,5-diaminonaphthalene
DHB: 2,5-dihydroxybenzoic acid
PC: phosphatidylcholine
Cer: ceramide
QC: quality control
GLs: glycerolipids
SPs: sphingolipids
GPs: glycerophospholipids
FC: fold change
pLSDA: partial least-squares discriminant analysis
SEM: standard error of the mean
pLSA: probabilistic latent semantic analysis
SM: sphingomyelin
SPH: sphingosine
LPCs: lysophosphatidylcholines
LPEs: lysophosphatidylethanolamines
LPA: lysophosphatidic acid
LPG: lysophosphatidylglycerol
LPI: lysophosphatidylinositol
PAs: phosphatidic acids
PEs: phosphatidylethanolamines
PGs: phosphatidylglycerols
PIs: phosphatidylinositols
PSs: phosphatidylserines
DGs: diacylglycerols
MGDG: monogalactosyldiacylglycerol
CmE: campesterol ester
ZyE: zymosteryl ester
SiE: sitosteryl ester
StE: stigmasteryl ester
acetyl-CoA: acetyl coenzyme A
Zy: zymosteryl
*Cm*: campesterol
Si: sitosteryl
St: stigmasteryl
