# Supplementary material for: Spatial Lipidomics Reveals Lipid Changes in the Cotyledon and Plumule of Mung Bean Seeds during Germination
Source: J Agric Food Chem. 2023 Nov 29;71(49):19879–87. doi: 10.1021/acs.jafc.3c06029 (PMC10722537; doi:10.1021/acs.jafc.3c06029)
Supplement: Supplementary file 1 — jf3c06029_si_001.pdf [file jf3c06029_si_001.pdf]

Spatial lipidomics reveals lipid changes in the cotyledon and plumule of mung bean seeds during germination

Peisi Xie[1$], Jing Chen[1$], Pengfei Wu[2,3], Zongwei Cai[1,2]*

[1]Ministry of Education Key Laboratory of Analytical Science for Food Safety and Biology, Fujian Provincial Key Laboratory of Analysis and Detection Technology for Food Safety, College of Chemistry, Fuzhou University, Fuzhou, Fujian, 350116, China

[2]State Key Laboratory of Environmental and Biological Analysis, Department of Chemistry, Hong Kong Baptist University, Hong Kong SAR 999077, China

[3]College of Forestry, Nanjing Forestry University, Nanjing, Jiangsu, 210018, China

[$]P. X. and J. C. contributed equally to this paper.

Figure S1. The loading plot of five internal structures in mung bean seeds.

Figure S2. Cross validations of pLSDA score plots.

Figure S3. Venn diagrams for various lipid classes.

Figure S4. Representative images of different lipid ion species in mung bean seeds.

Table S1. Information of lipid markers in the plumule between 24 h and 96 h identified by UPLC-MS/MS.

Table S2. Information of lipid markers in the cotyledon between 24 h and 96 h identified by UPLC-MS/MS.

Table S3. Information of significantly changed lipids in the cotyledon of mung bean seeds between germination day 1 and 4 identified by MALDI-MSI.

Table S4. Information of significantly changed lipids in the plumule of mung bean seeds between germination day 1 and 4 identified by MALDI-MSI.

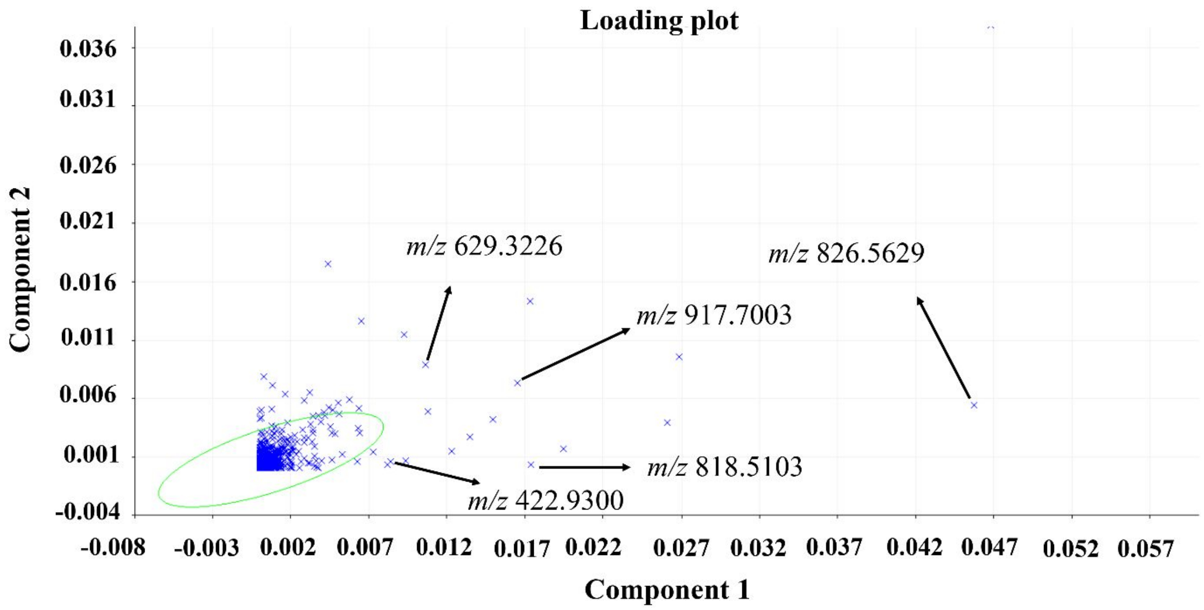

Figure S1 The loading plot of five internal structures in mung bean seeds. Selected ions locating out of 95% confidence ellipse were indicated by black arrows.

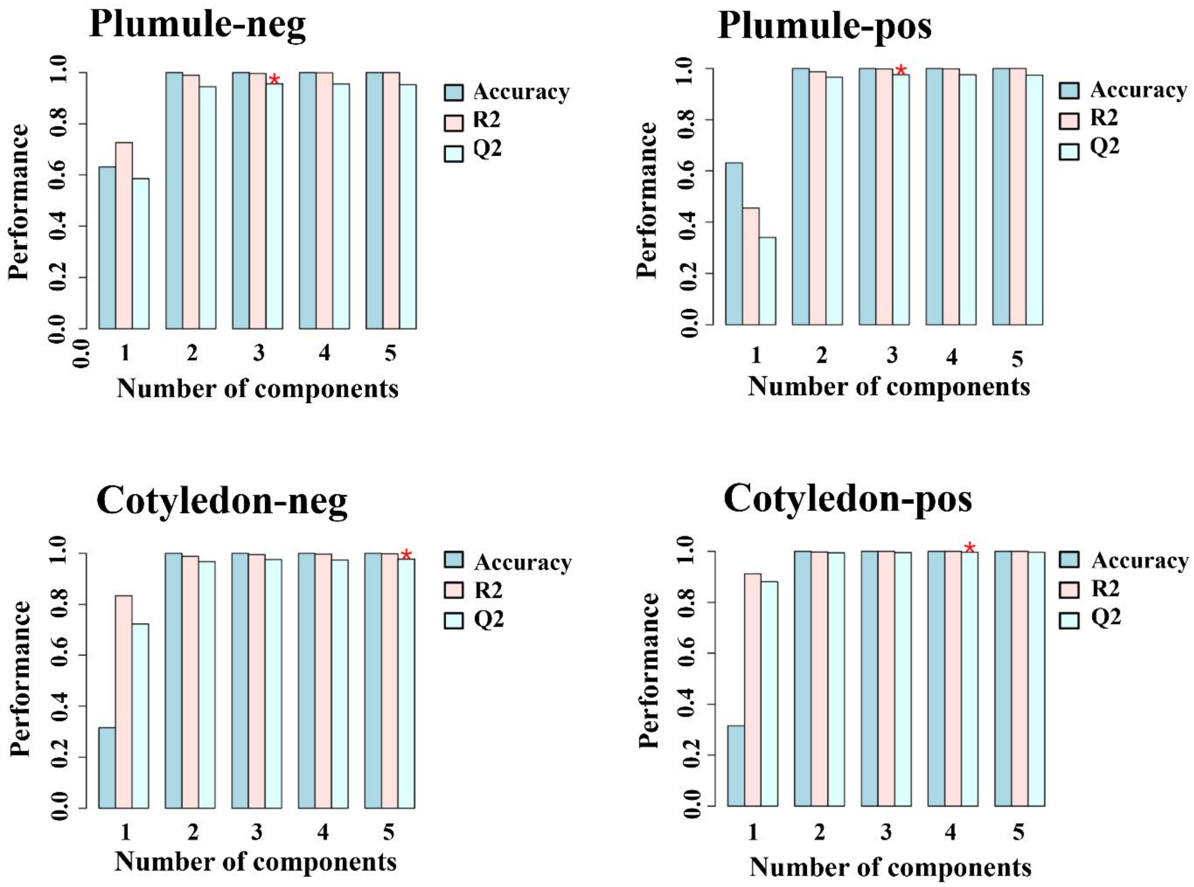

Figure S2. Cross validations of pLSDA score plots based on LC-MS-based lipidomic data of the plumule and cotyledon in mung bean seeds between germinating 24 and 96 h in negative and positive ionization modes.

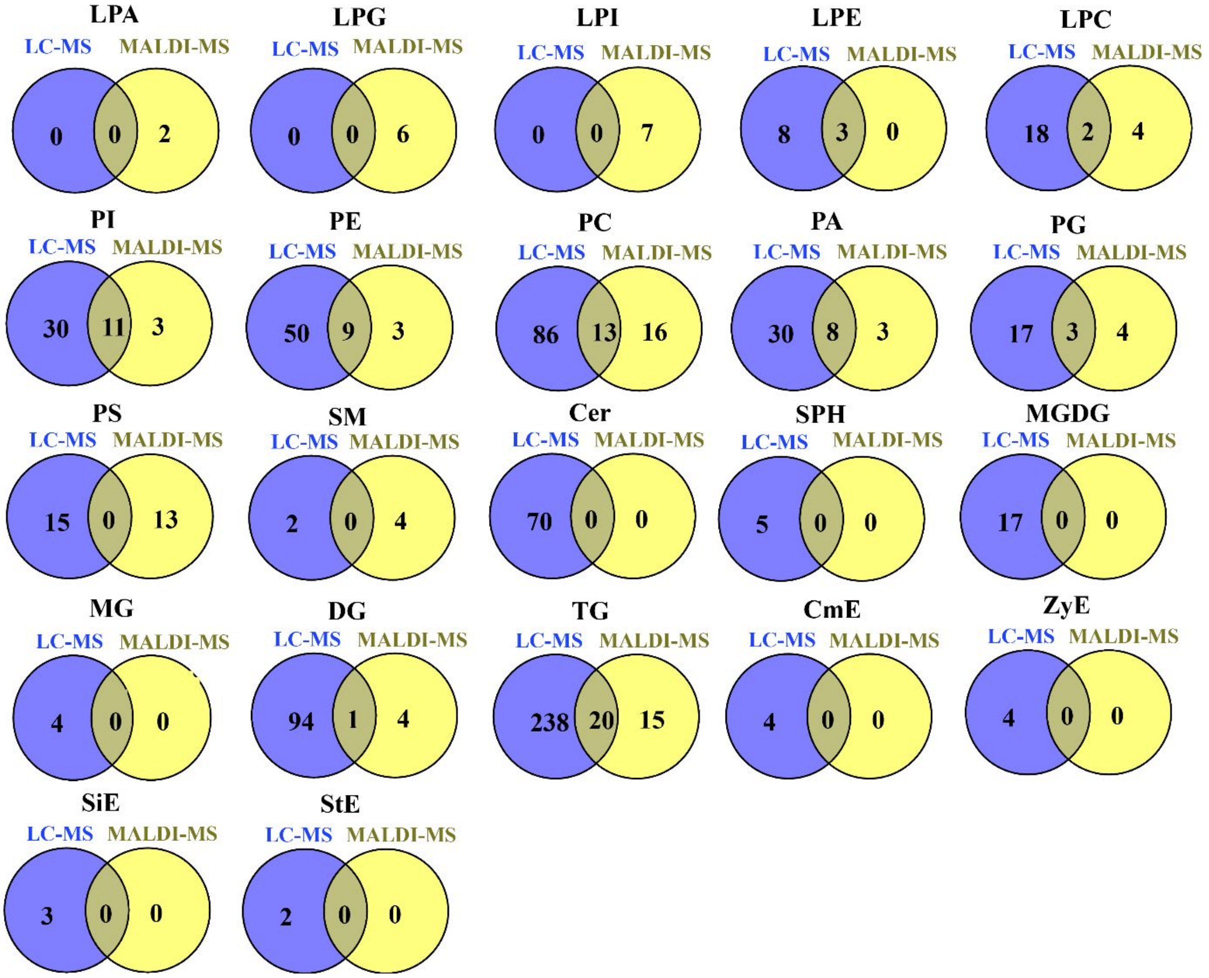

Figure S3. Venn diagrams for various lipid classes. The diagrams show the number of shared lipid species in different lipid classes between LC-MS and MALDI-MS.

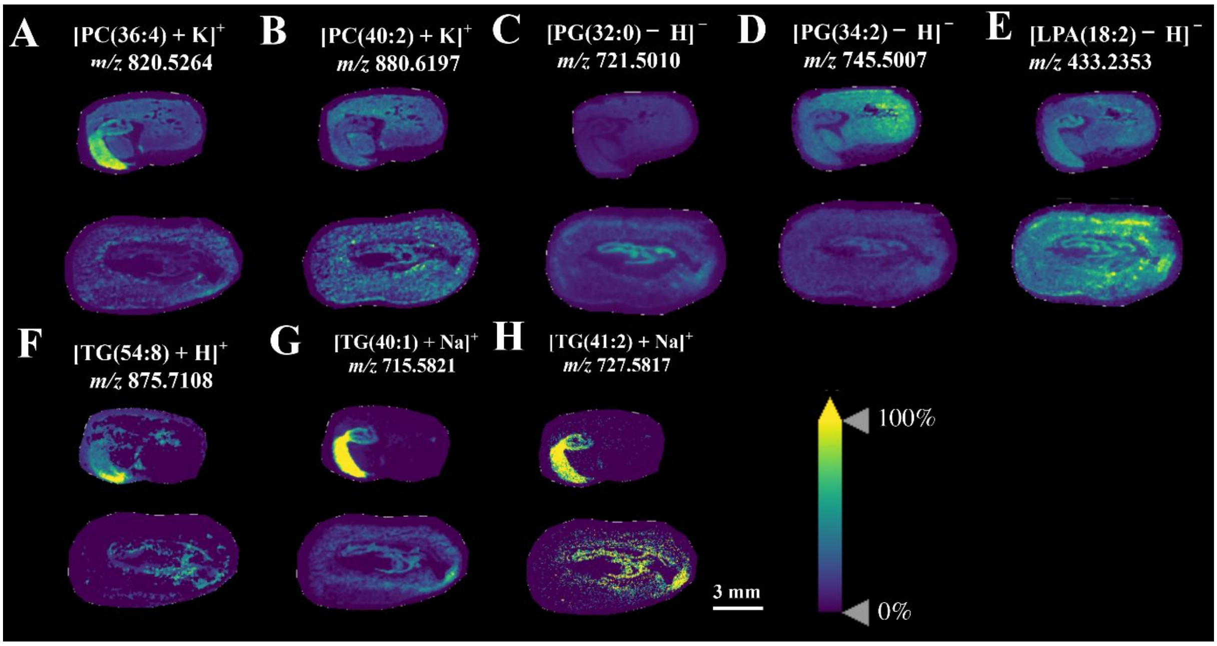

Figure S4. (A-H) Representative images of different lipid ion species in mung bean seeds between germinating 24 and 96 h. Scale bar = 3 mm for all seed sections.

Table S1. Information of lipid markers in the plumule between 24 h and 96 h identified by UPLC-MS/MS. The letter "P" and "N" refer to the "positive" and "negative" ionization modes, respectively.

No	Name	Detection mode	Fold change (96 h/24 h)	*p* value
1	Cer(d17:0_21:0)	N	1.63	0.0000
2	Cer(d17:1_21:0)	N	3.97	0.0000
3	Cer(d17:1_22:0)	N	1.65	0.0000
4	Cer(d17:1_23:0)	N	2.79	0.0016
5	Cer(d17:1_25:0)	N	1.96	0.0000
6	Cer(d18:0_16:0)	N	1.51	0.0023
7	Cer(d18:0_17:0)	P	1.72	0.0001
8	Cer(d18:0_21:0)	N	1.66	0.0000
9	Cer(d18:1_16:0)	N	0.62	0.0000
10	Cer(d18:1_26:0+O)	P	0.50	0.0005
11	Cer(d18:2_16:0)	P	0.65	0.0002
12	Cer(d18:2_16:1)	P	1.81	0.0022
13	Cer(d34:1)	N	0.59	0.0001
14	Cer(m18:0_16:0)	P	10.92	0.0000
15	Cer(m34:1)	P	0.28	0.0017
16	Cer(t16:0_18:1+O)	N	6.43	0.0000
17	Cer(t17:1_25:0+O)	N	2.88	0.0012
18	Cer(t18:0_16:0+O)	P	7.57	0.0000
19	Cer(t18:0_20:0)	P+N	0.36	0.0001

20	Cer(t18:0_21:0)	N	0.36	0.0000
21	Cer(t18:0_22:0+O)	P+N	1.83	0.0000
22	Cer(t18:0_23:0)	P+N	0.41	0.0000
23	Cer(t18:0_25:0)	P+N	0.26	0.0000
24	Cer(t18:0_25:0+O)	P	0.61	0.0000
25	Cer(t18:1_16:0)	P	1.69	0.0016
26	Cer(t18:1_22:0)	P+N	2.62	0.0000
27	Cer(t18:1_22:0+O)	P+N	3.14	0.0000
28	Cer(t18:1_24:0)	P+N	2.06	0.0012
29	Cer(t18:1_24:0+O)	P	3.14	0.0001
30	Cer(t18:1_25:0)	P+N	0.52	0.0000
31	Cer(t18:1_26:0)	N	1.78	0.0013
32	Cer(t18:1_26:0+O)	P	1.96	0.0000
33	CmE(18:2)	P	0.11	0.0000
34	CmE(18:3)	P	0.21	0.0000
35	CmE(20:3)	P	0.38	0.0000
36	CmE(20:4)	P	0.60	0.0000
37	DG(14:0_18:2)	P	0.01	0.0001
38	DG(14:0_18:3)	P	0.20	0.0000
39	DG(15:0_18:2)	P	0.03	0.0001
40	DG(16:0_16:0)	P	0.11	0.0002
41	DG(16:0_16:1)	P	0.05	0.0000
42	DG(16:0_18:1)	P	0.04	0.0000
43	DG(16:0_18:2)	P	0.04	0.0000

44	DG(16:0_18:3)	P	0.20	0.0001
45	DG(16:0_22:0)	P	0.10	0.0000
46	DG(17:0_18:1)	P	0.07	0.0001
47	DG(17:1_18:2)	P	0.05	0.0000
48	DG(17:1_18:3)	P	0.31	0.0000
49	DG(18:0_16:0)	P	0.15	0.0000
50	DG(18:0_18:0)	P	0.16	0.0000
51	DG(18:0_18:1)	P	0.04	0.0000
52	DG(18:0_18:3)	P	0.09	0.0001
53	DG(18:1_18:2)	P	0.03	0.0001
54	DG(18:1_22:0)	P	0.13	0.0000
55	DG(18:1_24:0)	P	0.22	0.0001
56	DG(18:2_18:2)	P	0.04	0.0001
57	DG(18:2_21:0)	P	0.01	0.0000
58	DG(18:3_18:2)	P	0.09	0.0001
59	DG(18:3_18:3)	P	0.24	0.0006
60	DG(18:3_21:0)	P	0.04	0.0000
61	DG(18:3_23:0)	P	0.04	0.0000
62	DG(18:4_16:0)	P	0.22	0.0000
63	DG(18:4_18:1)	P	0.04	0.0001
64	DG(18:4_18:2)	P	0.13	0.0001
65	DG(19:1_18:2)	P	0.11	0.0004
66	DG(19:1_18:3)	P	0.32	0.0000
67	DG(20:0_18:3)	P	0.05	0.0002

68	DG(20:0_22:0)	P	0.34	0.0019
69	DG(20:1_18:1)	P	0.05	0.0002
70	DG(20:1_18:2)	P	0.03	0.0001
71	DG(20:3_18:2)	P	0.10	0.0000
72	DG(22:0_18:2)	P	0.04	0.0000
73	DG(22:0_18:3)	P	0.11	0.0000
74	DG(22:0_24:0)	P	85.56	0.0013
75	DG(24:0_18:2)	P	0.08	0.0000
76	DG(24:0_18:3)	P	0.19	0.0001
77	DG(24:0_23:0)	P	44.56	0.0006
78	DG(24:1_18:2)	P	0.03	0.0001
79	DG(24:1_18:3)	P	0.16	0.0001
80	DG(25:0_18:2)	P	0.04	0.0000
81	DG(26:0_18:1)	P	0.47	0.0008
82	DG(26:0_18:2)	P	0.31	0.0000
83	DG(26:0_24:1)	P	10.64	0.0015
84	DG(29:0)	P	50.89	0.0004
85	DG(30:0e)	P	0.04	0.0001
86	DG(30:1e)	P	0.02	0.0001
87	DG(31:0e)	P	0.11	0.0001
88	DG(31:1)	P	107.10	0.0007
89	DG(31:1e)	P	0.04	0.0001
90	DG(31:3)	P	271.30	0.0007
91	DG(32:0e)	P	0.08	0.0001

92	DG(32:1e)	P	0.04	0.0000
93	DG(32:2e)	P	0.54	0.0038
94	DG(32:3e)	P	0.04	0.0001
95	DG(33:1e)	P	0.09	0.0000
96	DG(33:2e)	P	0.36	0.0005
97	DG(33:3)	P	94.20	0.0001
98	DG(33:4)	P	9.46	0.0024
99	DG(34:1e)	P	0.08	0.0032
100	DG(34:2e)	P	0.05	0.0000
101	DG(34:3e)	P	0.07	0.0000
102	DG(34:4e)	P	0.12	0.0002
103	DG(34:5e)	P	0.17	0.0001
104	DG(36:1e)	P	0.03	0.0004
105	DG(36:2e)	P	0.06	0.0001
106	DG(36:3e)	P	0.05	0.0003
107	DG(36:4e)	P	0.04	0.0001
108	DG(36:5e)	P	0.03	0.0000
109	DG(38:1e)	P	0.08	0.0000
110	DG(38:3e)	P	0.04	0.0002
111	LPC(17:0)	P	2.52	0.0000
112	LPC(18:2)	P	0.05	0.0077
113	LPC(20:0)	P+N	2.81	0.0002
114	LPC(20:1)	P	4.56	0.0000
115	LPC(20:3)	P	2.65	0.0000

116	LPC(22:0)	P+N	7.88	0.0002
117	LPC(22:1)	P	9.44	0.0000
118	LPC(22:3)	P	3.34	0.0001
119	LPC(24:0)	P+N	18.00	0.0000
120	LPC(24:1)	P	12.00	0.0004
121	LPC(26:0)	P	26.70	0.0000
122	LPC(26:1)	P	29.40	0.0000
123	LPC(28:0)	P	7.29	0.0000
124	LPE(16:0)	P+N	2.51	0.0000
125	LPE(18:0)	P+N	3.75	0.0000
126	LPE(20:0)	N	5.33	0.0000
127	LPE(22:0)	P+N	13.02	0.0000
128	LPE(23:0)	N	5.05	0.0000
129	LPE(24:0)	N	55.09	0.0000
130	LPE(24:1)	N	18.56	0.0000
131	MG(16:0)	P	0.23	0.0000
132	MGDG(16:0_18:0)	N	0.28	0.0002
133	MGDG(16:0_18:1)	N	0.31	0.0000
134	MGDG(16:0_18:2)	N	0.21	0.0000
135	MGDG(16:0_18:3)	N	2.15	0.0000
136	MGDG(16:0_29:10)	N	4.46	0.0000
137	MGDG(18:0_18:1)	N	0.15	0.0000
138	MGDG(18:0_29:10)	N	2.05	0.0000
139	MGDG(18:1_18:1)	N	0.13	0.0000

140	MGDG(18:1_18:2)	N	0.08	0.0000
141	MGDG(18:2_18:2)	N	0.21	0.0000
142	MGDG(18:2_18:3)	N	0.33	0.0000
143	MGDG(18:3_18:3)	N	4.86	0.0000
144	MGDG(18:3_29:10)	N	4.83	0.0000
145	PA(16:0_16:0)	P+N	3.73	0.0018
146	PA(16:0_18:3)	P	3.55	0.0011
147	PA(17:1_18:3)	N	92.31	0.0000
148	PA(18:0_18:3)	P+N	2.83	0.0007
149	PA(18:1_18:1)	P	2.38	0.0002
150	PA(18:1_18:2)	P	0.65	0.0006
151	PA(18:3_18:2)	N	2.62	0.0001
152	PA(18:3_18:3)	P+N	7.78	0.0006
153	PA(20:0_18:2)	N	0.46	0.0001
154	PA(20:1_18:3)	P+N	8.04	0.0001
155	PA(22:0_18:3)	N	5.51	0.0008
156	PA(24:0_18:2)	P	1.62	0.0038
157	PA(24:0_18:3)	N	10.54	0.0000
158	PC(14:0_22:4)	P	0.08	0.0000
159	PC(16:0_18:1)	N	0.28	0.0000
160	PC(16:0_18:2)	N	0.14	0.0000
161	PC(16:0_18:3)	N	0.44	0.0000
162	PC(17:0_18:2)	P	0.21	0.0001
163	PC(17:1_18:2)	P	2.39	0.0007

164	PC(17:1_18:3)	P	0.25	0.0000
165	PC(18:0_18:1)	N	0.30	0.0000
166	PC(18:0_18:2)	N	0.26	0.0000
167	PC(18:0_18:3)	P+N	0.52	0.0039
168	PC(18:1_18:2)	N	0.13	0.0000
169	PC(18:1_21:1)	P	0.07	0.0005
170	PC(18:2_18:2)	N	0.07	0.0000
171	PC(18:3_18:2)	N	0.22	0.0000
172	PC(18:3_18:3)	N	0.46	0.0000
173	PC(19:1_16:0)	P	0.24	0.0000
174	PC(19:1_17:0)	P	0.31	0.0000
175	PC(19:1_18:1)	P	0.06	0.0000
176	PC(19:1_18:3)	P	0.24	0.0019
177	PC(20:0_18:2)	N	0.07	0.0000
178	PC(20:1_18:2)	P+N	0.17	0.0001
179	PC(22:0_14:1)	P	0.29	0.0000
180	PC(23:0e)	P	5.19	0.0002
181	PC(24:3e)	P	9.69	0.0006
182	PC(25:0e)	P	29.90	0.0000
183	PC(33:1)	P	0.26	0.0001
184	PC(33:3)	P	0.34	0.0000
185	PC(34:1)	P	0.23	0.0000
186	PC(34:2)	P	0.19	0.0000
187	PC(34:3)	P	0.53	0.0000

188	PC(35:5)	P	0.28	0.0001
189	PC(36:0)	P	0.60	0.0007
190	PC(36:2)	P	0.20	0.0000
191	PC(36:3)	P	0.17	0.0001
192	PC(36:4)	P	0.16	0.0002
193	PC(36:5)	P	0.16	0.0001
194	PC(36:6)	P	0.51	0.0005
195	PC(38:1)	P	0.35	0.0006
196	PC(38:2)	P	0.06	0.0001
197	PC(38:3)	P	0.17	0.0000
198	PC(38:5)	P	0.22	0.0001
199	PC(38:9)	P	0.13	0.0003
200	PC(40:0)	P	1.70	0.0000
201	PC(41:1)	P	0.59	0.0076
202	PC(44:1)	P	6.35	0.0038
203	PE(16:0_18:1)	P+N	0.60	0.0000
204	PE(16:0_18:2)	P+N	0.46	0.0000
205	PE(16:0_18:3)	P+N	3.13	0.0000
206	PE(18:0_18:1)	P+N	0.51	0.0000
207	PE(18:1_18:1)	P+N	0.35	0.0000
208	PE(18:1_18:2)	P+N	0.29	0.0000
209	PE(18:1_22:0)	N	2.16	0.0000
210	PE(18:1_24:0)	P+N	3.71	0.0001
211	PE(18:2_18:2)	P+N	0.22	0.0000

212	PE(18:2_23:0)	N	0.56	0.0001
213	PE(18:3_18:2)	P+N	0.57	0.0000
214	PE(18:3_18:3)	P+N	2.37	0.0000
215	PE(20:0_18:2)	N	0.45	0.0000
216	PE(20:1_18:2)	N	0.56	0.0000
217	PE(20:3_22:3)	P	19.50	0.0003
218	PE(22:0_18:3)	P	3.32	0.0000
219	PE(24:0_18:2)	P+N	1.71	0.0055
220	PE(24:0_18:3)	P+N	10.69	0.0001
221	PE(26:0_18:2)	N	3.62	0.0001
222	PE(26:0_18:3)	N	16.84	0.0000
223	PE(34:0)	P	0.24	0.0000
224	PE(34:3)	P	4.16	0.0007
225	PE(36:4)	P	0.40	0.0008
226	PE(36:5)	P	0.52	0.0002
227	PE(38:1)	P	3.57	0.0000
228	PE(38:4)	P	2.69	0.0000
229	PE(40:3)	P	0.25	0.0001
230	PE(40:4)	P	3.59	0.0002
231	PE(40:6)	P	2.58	0.0030
232	PE(41:2)	P	0.59	0.0003
233	PE(41:3)	P	1.80	0.0001
234	PE(42:3)	P	0.16	0.0000
235	PE(42:4)	P	1.95	0.0002

236	PE(55:3)	P	11.60	0.0003
237	PG(16:0_16:0)	P+N	4.98	0.0000
238	PG(16:0_17:0)	N	11.85	0.0010
239	PG(16:0_18:2)	P+N	0.48	0.0000
240	PG(16:0_18:3)	P+N	4.87	0.0000
241	PG(18:0_16:0)	P+N	2.13	0.0091
242	PG(18:0_18:2)	P+N	0.46	0.0000
243	PG(18:0_18:3)	N	3.31	0.0000
244	PG(18:2_18:2)	P+N	0.09	0.0022
245	PG(18:3_18:2)	N	0.54	0.0000
246	PG(18:3_18:3)	N	3.19	0.0000
247	PG(32:0)	P	36.30	0.0001
248	PG(33:0)	P	16.90	0.0001
249	PG(34:0)	P	6.43	0.0000
250	PG(36:0)	P	0.62	0.0051
251	PG(36:3)	P	0.25	0.0000
252	PG(36:6)	N	1.85	0.0000
253	PI(16:0_16:0)	P+N	0.36	0.0000
254	PI(16:0_18:1)	P+N	0.32	0.0000
255	PI(16:0_18:2)	P+N	0.23	0.0000
256	PI(16:0_18:3)	P	0.62	0.0004
257	PI(17:0_18:1)	N	0.64	0.0000
258	PI(17:0_18:2)	N	0.30	0.0000
259	PI(17:0_18:3)	N	2.81	0.0000

260	PI(18:0_16:0)	N	0.33	0.0001
261	PI(18:0_18:1)	N	0.31	0.0000
262	PI(18:0_18:2)	N	0.20	0.0000
263	PI(18:0_18:3)	P+N	0.66	0.0000
264	PI(18:1_18:1)	P	0.13	0.0000
265	PI(18:1_18:2)	P+N	0.24	0.0000
266	PI(18:2_18:2)	P+N	0.10	0.0014
267	PI(18:2_23:0)	N	0.04	0.0000
268	PI(18:3_18:2)	P+N	0.37	0.0000
269	PI(20:0_18:2)	N	0.11	0.0000
270	PI(22:0_18:2)	N	0.09	0.0000
271	PI(24:0_18:2)	N	0.11	0.0000
272	PI(24:0_18:3)	N	0.56	0.0000
273	PI(35:2)	P	0.25	0.0000
274	PI(36:1)	P	0.18	0.0000
275	PI(36:3)	P	0.15	0.0000
276	PI(36:5)	P	0.27	0.0000
277	PI(38:1)	P	3.47	0.0000
278	PI(42:2)	P	0.14	0.0000
279	PS(14:0_20:2)	P	0.24	0.0001
280	PS(22:0_18:2)	N	0.30	0.0001
281	PS(24:0_18:3)	N	6.80	0.0018
282	PS(38:2)	P	0.22	0.0000
283	PS(38:6)	N	0.28	0.0000

284	PS(40:2)	P	0.28	0.0000
285	PS(42:3)	P	3.16	0.0000
286	SM(d32:4)	P	0.26	0.0004
287	SPH(t18:0)	P	0.18	0.0017
288	SiE(18:1)	P	0.08	0.0000
289	SiE(18:2)	P	0.13	0.0000
290	SiE(18:3)	P	0.26	0.0000
291	StE(18:2)	P	0.11	0.0000
292	StE(18:3)	P	0.15	0.0000
293	TG(10:0_10:2_18:1)	P	0.15	0.0004
294	TG(12:0_18:2_18:2)	P	0.03	0.0000
295	TG(12:0_18:2_18:3)	P	0.03	0.0002
296	TG(12:0e_10:3_11:3)	P	44.10	0.0009
297	TG(13:0_10:3_10:3)	P	11.70	0.0034
298	TG(14:0_18:2_18:2)	P	0.07	0.0000
299	TG(14:0_18:2_18:3)	P	0.05	0.0000
300	TG(14:0_18:3_18:3)	P	0.08	0.0001
301	TG(15:0_15:0_15:0)	P	0.32	0.0001
302	TG(15:0_16:0_16:0)	P	0.09	0.0001
303	TG(15:0_16:0_18:2)	P	0.03	0.0001
304	TG(15:0_16:0_18:3)	P	0.04	0.0002
305	TG(15:0_16:1_18:3)	P	0.04	0.0003
306	TG(15:0_18:1_18:1)	P	0.03	0.0000
307	TG(15:0_18:1_18:2)	P	0.04	0.0000

308	TG(15:0_18:2_18:2)	P	0.04	0.0002
309	TG(15:0_18:2_18:3)	P	0.04	0.0001
310	TG(15:0_18:3_18:3)	P	0.06	0.0001
311	TG(16:0_12:0_18:2)	P	0.05	0.0004
312	TG(16:0_12:0_18:3)	P	0.07	0.0002
313	TG(16:0_14:0_16:0)	P	0.25	0.0036
314	TG(16:0_14:0_18:1)	P	0.10	0.0001
315	TG(16:0_14:0_18:2)	P	0.06	0.0001
316	TG(16:0_14:0_22:6)	P	0.01	0.0001
317	TG(16:0_16:0_16:0)	P	0.07	0.0006
318	TG(16:0_16:0_17:0)	P	0.06	0.0003
319	TG(16:0_16:0_18:1)	P	0.08	0.0003
320	TG(16:0_16:0_18:2)	P	0.05	0.0002
321	TG(16:0_16:0_18:3)	P	0.07	0.0000
322	TG(16:0_16:0_21:0)	P	0.03	0.0005
323	TG(16:0_16:0_22:0)	P	0.04	0.0003
324	TG(16:0_16:0_23:0)	P	0.03	0.0002
325	TG(16:0_16:0_24:0)	P	0.03	0.0002
326	TG(16:0_17:0_18:1)	P	0.03	0.0001
327	TG(16:0_18:1_18:1)	P	0.06	0.0001
328	TG(16:0_18:1_18:2)	P	0.05	0.0001
329	TG(16:0_18:1_21:0)	P	0.03	0.0001
330	TG(16:0_18:1_22:0)	P	0.04	0.0002
331	TG(16:0_18:1_23:0)	P	0.03	0.0001

332	TG(16:0_18:1_24:0)	P	0.04	0.0000
333	TG(16:0_18:2_18:2)	P	0.07	0.0000
334	TG(16:0_18:2_21:0)	P	0.02	0.0000
335	TG(16:0_18:3_18:3)	P	0.14	0.0000
336	TG(16:0_6:0_12:0)	P	0.11	0.0001
337	TG(16:0_6:0_12:1)	P	0.18	0.0000
338	TG(16:0_6:0_12:2)	P	0.34	0.0001
339	TG(16:0_8:0_10:2)	P	0.26	0.0000
340	TG(16:0_8:0_10:3)	P	9.59	0.0022
341	TG(16:1_18:1_20:5)	P	0.00	0.0002
342	TG(16:1_18:2_18:2)	P	0.07	0.0001
343	TG(16:1_18:3_18:3)	P	0.18	0.0002
344	TG(17:0_18:1_18:1)	P	0.03	0.0001
345	TG(17:0_18:1_18:2)	P	0.03	0.0000
346	TG(18:0_16:0_16:0)	P	0.06	0.0001
347	TG(18:0_16:0_17:0)	P	0.05	0.0001
348	TG(18:0_16:0_18:1)	P	0.05	0.0000
349	TG(18:0_16:0_23:0)	P	0.03	0.0002
350	TG(18:0_17:0_18:1)	P	0.03	0.0000
351	TG(18:0_18:0_18:1)	P	0.05	0.0001
352	TG(18:0_18:1_18:1)	P	0.04	0.0000
353	TG(18:1_18:1_18:1)	P	0.05	0.0000
354	TG(18:1_18:1_18:2)	P	0.03	0.0047
355	TG(18:1_18:1_20:4)	P	0.40	0.0021

356	TG(18:1_18:1_21:0)	P	0.02	0.0001
357	TG(18:1_18:1_22:0)	P	0.04	0.0001
358	TG(18:1_18:1_22:1)	P	0.04	0.0000
359	TG(18:1_18:1_23:0)	P	0.02	0.0001
360	TG(18:1_18:1_24:0)	P	0.03	0.0000
361	TG(18:1_18:2_18:2)	P	0.06	0.0004
362	TG(18:1_18:2_22:1)	P	0.05	0.0000
363	TG(18:1_18:2_23:0)	P	0.03	0.0001
364	TG(18:1_18:2_24:0)	P	0.03	0.0001
365	TG(18:2_18:2_18:2)	P	0.06	0.0001
366	TG(18:2_18:2_23:0)	P	0.03	0.0000
367	TG(18:3_13:0_20:4)	P	0.07	0.0003
368	TG(18:3_14:1_18:3)	P	0.01	0.0033
369	TG(18:3_17:1_18:3)	P	0.09	0.0001
370	TG(18:3_18:2_18:2)	P	0.08	0.0000
371	TG(18:3_18:2_18:3)	P	0.09	0.0001
372	TG(18:3_18:2_23:0)	P	0.04	0.0001
373	TG(18:3_18:3_18:3)	P	0.28	0.0001
374	TG(18:3_18:3_20:2)	P	0.10	0.0000
375	TG(18:3_18:3_20:3)	P	0.30	0.0012
376	TG(18:3_18:3_20:5)	P	0.25	0.0000
377	TG(18:3_18:3_21:0)	P	0.21	0.0000
378	TG(18:3_18:3_22:1)	P	0.22	0.0001
379	TG(18:3_18:3_22:2)	P	0.21	0.0000

380	TG(18:3_18:3_23:0)	P	0.20	0.0002
381	TG(18:3_18:3_23:1)	P	0.18	0.0000
382	TG(18:4_16:0_18:3)	P	0.55	0.0039
383	TG(18:4_18:1_18:1)	P	0.05	0.0000
384	TG(19:1_18:1_18:1)	P	0.04	0.0008
385	TG(19:1_18:1_18:2)	P	0.04	0.0000
386	TG(19:1_18:2_18:3)	P	0.08	0.0005
387	TG(20:0_16:0_16:0)	P	0.06	0.0011
388	TG(20:0_18:1_18:1)	P	0.04	0.0001
389	TG(20:1_18:1_18:1)	P	0.04	0.0003
390	TG(20:1_18:1_18:2)	P	0.06	0.0001
391	TG(20:1_18:2_18:2)	P	0.07	0.0003
392	TG(20:1_18:2_18:3)	P	0.08	0.0000
393	TG(20:1_18:3_18:3)	P	0.15	0.0001
394	TG(20:2e_18:2_18:3)	P	0.01	0.0031
395	TG(20:5_18:2_18:2)	P	0.18	0.0000
396	TG(22:0_18:2_18:3)	P	0.07	0.0001
397	TG(22:0_18:3_18:3)	P	0.12	0.0000
398	TG(24:0_18:2_18:2)	P	0.04	0.0001
399	TG(24:0_18:2_18:3)	P	0.06	0.0000
400	TG(24:0_18:3_18:3)	P	0.13	0.0000
401	TG(24:1_18:2_18:2)	P	0.06	0.0001
402	TG(24:1_18:2_18:3)	P	0.11	0.0002
403	TG(24:1_18:3_18:3)	P	0.20	0.0001

404	TG(25:0_16:0_18:1)	P	0.03	0.0001
405	TG(25:0_18:0_18:1)	P	0.03	0.0000
406	TG(25:0_18:1_18:1)	P	0.03	0.0001
407	TG(25:0_18:1_18:2)	P	0.02	0.0001
408	TG(25:0_18:2_18:2)	P	0.03	0.0002
409	TG(25:0_18:2_18:3)	P	0.04	0.0001
410	TG(25:0_18:3_18:3)	P	0.11	0.0001
411	TG(26:0_16:0_18:1)	P	0.03	0.0000
412	TG(26:0_18:1_18:1)	P	0.03	0.0000
413	TG(26:0_18:1_18:2)	P	0.03	0.0000
414	TG(26:0_18:2_18:2)	P	0.04	0.0001
415	TG(26:0_18:2_18:3)	P	0.04	0.0003
416	TG(26:0_18:3_18:3)	P	0.11	0.0000
417	TG(26:1_18:2_18:2)	P	0.04	0.0002
418	TG(26:1_18:3_18:3)	P	0.21	0.0012
419	TG(27:0_18:0_18:1)	P	0.05	0.0000
420	TG(27:0_18:1_18:1)	P	0.03	0.0000
421	TG(27:0_18:1_18:2)	P	0.03	0.0000
422	TG(27:0_18:2_18:2)	P	0.03	0.0000
423	TG(27:0_18:2_18:3)	P	0.03	0.0001
424	TG(27:0_18:3_18:3)	P	0.10	0.0002
425	TG(28:0_16:0_16:0)	P	0.06	0.0002
426	TG(28:0_18:0_18:1)	P	0.08	0.0000
427	TG(28:0_18:1_18:1)	P	0.04	0.0000

428	TG(28:0_18:1_18:2)	P	0.03	0.0000
429	TG(28:0_18:2_18:3)	P	0.03	0.0001
430	TG(28:0_18:3_18:3)	P	0.08	0.0000
431	TG(28:1_18:1_18:2)	P	0.01	0.0000
432	TG(28:1_18:2_18:3)	P	0.05	0.0001
433	TG(28:1_18:3_18:3)	P	0.15	0.0001
434	TG(29:0_18:1_18:2)	P	0.04	0.0000
435	TG(29:0_18:2_18:2)	P	0.04	0.0000
436	TG(29:0_18:2_18:3)	P	0.04	0.0000
437	TG(29:0_18:2_21:1)	P	0.00	0.0000
438	TG(29:0_18:3_18:3)	P	0.10	0.0001
439	TG(30:0_18:1_18:1)	P	0.07	0.0000
440	TG(30:0_18:2_18:3)	P	0.05	0.0000
441	TG(30:0_18:3_18:3)	P	0.09	0.0001
442	TG(30:1_18:1_18:2)	P	0.05	0.0000
443	TG(30:1_18:1_24:2)	P	0.03	0.0001
444	TG(30:1_18:2_18:2)	P	0.04	0.0000
445	TG(30:1_18:2_18:3)	P	0.05	0.0002
446	TG(30:1_18:2_20:1)	P	0.01	0.0066
447	TG(30:1_18:3_18:3)	P	0.12	0.0003
448	TG(36:4)	P	0.18	0.0000
449	TG(38:3)	P	0.01	0.0030
450	TG(38:5)	P	0.00	0.0000
451	TG(38:6)	P	0.04	0.0002

452	TG(39:6)	P	0.30	0.0001
453	TG(4:0_14:3_16:0)	P	0.08	0.0000
454	TG(4:0_14:3_18:1)	P	0.13	0.0001
455	TG(4:0_16:1_18:1)	P	0.01	0.0026
456	TG(4:0_18:2_18:2)	P	0.00	0.0002
457	TG(41:7)	P	0.03	0.0001
458	TG(43:7)	P	0.01	0.0001
459	TG(43:8)	P	0.02	0.0004
460	TG(44:8)	P	0.01	0.0003
461	TG(46:10)	P	0.01	0.0003
462	TG(49:7)	P	0.08	0.0003
463	TG(6:0_12:2_18:2)	P	0.05	0.0034
464	TG(6:0_14:2_18:2)	P	0.24	0.0003
465	TG(6:0_14:3_18:2)	P	0.47	0.0056
466	TG(67:5)	P	0.06	0.0000
467	TG(70:7)	P	0.07	0.0001
468	TG(72:6)	P	0.03	0.0001
469	TG(8:0_10:3_13:0)	P	121.00	0.0013
470	ZyE(11:0)	P	0.04	0.0000
471	ZyE(19:0)	P	1.79	0.0037
472	ZyE(22:2)	P	0.12	0.0000
473	ZyE(22:3)	P	0.23	0.0000

Table S2. Information of lipid markers in the cotyledon between 24 h and 96 h identified by UPLC-MS/MS. The letter "P" and "N" refer to the "positive" and "negative" ionization modes, respectively.

No	Name	Detection mode	Fold change (96 h/24 h)	*p* value
1	Cer(d17:0_21:0)	N	0.550	0.0000
2	Cer(d17:1_21:0)	N	1.950	0.0009
3	Cer(d17:1_23:0)	N	3.290	0.0007
4	Cer(d18:0_22:0)	P	0.403	0.0000
5	Cer(d18:0_24:0)	P	0.524	0.0001
6	Cer(d32:1)	P	0.584	0.0009
7	Cer(d33:1)	P	0.595	0.0090
8	Cer(d34:1)	P	0.560	0.0069
9	Cer(d36:1)	P	5.180	0.0010
10	Cer(t17:0_23:0+O)	N	0.532	0.0000
11	Cer(t18:0_20:0)	P+N	0.133	0.0000
12	Cer(t18:0_21:0)	N	0.140	0.0000
13	Cer(t18:0_21:0+O)	P	0.359	0.0000
14	Cer(t18:0_22:0)	P+N	0.220	0.0000
15	Cer(t18:0_22:0+O)	P	0.564	0.0001
16	Cer(t18:0_23:0)	P+N	0.271	0.0000
17	Cer(t18:0_23:0+O)	P	0.508	0.0000
18	Cer(t18:0_24:0)	P+N	0.401	0.0000
19	Cer(t18:0_25:0)	P+N	0.386	0.0000

20	Cer(t18:1_23:0)	P+N	2.260	0.0001
21	Cer(t18:1_24:0)	P+N	3.910	0.0000
22	Cer(t18:1_24:0+O)	P	2.820	0.0012
23	Cer(t18:1_25:0)	P+N	5.870	0.0001
24	Cer(t18:1_26:0)	P+N	13.100	0.0001
25	Cer(t18:1_26:0+O)	P	6.450	0.0004
26	Cer(t39:0+O)	N	0.327	0.0000
27	Cer(t41:0+O)	N	0.577	0.0000
28	CmE(18:3)	P	3.87	0.001
29	DG(16:0_18:2)	P+N	0.540	0.0015
30	DG(16:0_18:3)	P+N	0.479	0.0031
31	DG(16:0_22:0)	P	1.530	0.0076
32	DG(16:1_18:3)	P	3.740	0.0002
33	DG(18:0_18:3)	P+N	0.573	0.0001
34	DG(18:1_18:2)	P	0.303	0.0002
35	DG(18:1_18:3)	P	0.360	0.0017
36	DG(18:1_22:0)	P	1.600	0.0001
37	DG(18:1_24:0)	P	2.160	0.0000
38	DG(18:2_18:2)	P	0.567	0.0006
39	DG(18:3_18:2)	P	0.660	0.0002
40	DG(18:3_18:3)	P	2.880	0.0000
41	DG(18:3_23:0)	P	0.559	0.0000
42	DG(18:4_16:0)	P	0.575	0.0013
43	DG(20:0_22:0)	P	4.140	0.0000

44	DG(22:0_18:3)	P	0.596	0.0002
45	DG(22:0_22:0)	P	5.610	0.0000
46	DG(22:0_24:0)	P	12.000	0.0000
47	DG(25:0_18:2)	P	3.940	0.0010
48	DG(26:0_18:2)	P	6.080	0.0011
49	DG(26:0_18:3)	P	4.730	0.0053
50	DG(30:1e)	P	0.139	0.0000
51	DG(31:2e)	P	0.175	0.0000
52	DG(32:0e)	P	0.150	0.0000
53	DG(32:1e)	P	0.368	0.0008
54	DG(33:1e)	P	0.421	0.0000
55	DG(34:1e)	P	0.434	0.0006
56	DG(34:2e)	P	0.144	0.0000
57	DG(34:3e)	P	0.149	0.0000
58	DG(34:4e)	P	0.171	0.0000
59	DG(34:5e)	P	0.239	0.0002
60	DG(36:4e)	P	0.547	0.0001
61	DG(40:2e)	P	0.441	0.0000
62	DG(51:1)	P	0.393	0.0003
63	LPC(16:0)	P	5.421	0.0000
64	LPC(17:0)	P	4.364	0.0000
65	LPC(18:0)	P+N	11.361	0.0003
66	LPC(20:0)	P+N	12.275	0.0008
67	LPC(20:1)	P	13.262	0.0002

68	LPC(20:3)	P	11.865	0.0001
69	LPC(20:5)	P	0.134	0.0087
70	LPC(22:0)	P+N	8.166	0.0003
71	LPC(22:3)	P	13.269	0.0002
72	LPC(24:0)	P+N	19.845	0.0004
73	LPC(26:0)	P	189.935	0.0007
74	LPE(16:0)	P+N	8.890	0.0007
75	LPE(18:0)	N	14.500	0.0000
76	LPE(18:2)	P	5.770	0.0002
77	LPE(18:3)	N	3.380	0.0000
78	LPE(20:0)	N	19.900	0.0000
79	LPE(22:0)	N	8.090	0.0000
80	LPE(23:0)	N	15.100	0.0000
81	LPE(24:0)	N	37.600	0.0000
82	MG(18:3)	P	1.570	0.0057
83	MGDG(16:0_18:2)	N	0.255	0.0000
84	MGDG(16:0_18:3)	N	22.000	0.0000
85	MGDG(18:0_18:2)	N	0.232	0.0000
86	MGDG(18:2_18:2)	N	0.136	0.0000
87	MGDG(18:2_18:3)	N	0.308	0.0000
88	MGDG(18:3_18:3)	N	6.340	0.0000
89	PA(15:0_18:2)	P+N	1.920	0.0000
90	PA(16:0_16:0)	N	1.590	0.0005
91	PA(16:0_18:3)	N	1.540	0.0002

92	PA(18:0_18:2)	P+N	1.710	0.0000
93	PA(18:0_18:3)	P+N	3.090	0.0020
94	PA(18:1_18:2)	P	0.216	0.0000
95	PA(18:2_18:2)	P+N	1.640	0.0002
96	PA(22:0_18:2)	N	0.519	0.0038
97	PA(24:0_18:2)	P+N	2.560	0.0051
98	PA(34:2)	P	3.580	0.0003
99	PA(34:3)	P	6.700	0.0000
100	PA(36:3)	P	7.970	0.0000
101	PA(36:5)	P	7.020	0.0080
102	PA(36:6)	P	2.000	0.0000
103	PC(14:0_18:2)	N	0.227	0.0003
104	PC(14:0_22:4)	P	0.332	0.0007
105	PC(15:0_18:2)	N	0.442	0.0000
106	PC(16:0_16:0)	N	0.267	0.0001
107	PC(16:0_18:1)	N	0.204	0.0003
108	PC(16:0_18:2)	N	0.391	0.0008
109	PC(16:0_18:3)	N	0.380	0.0002
110	PC(16:1_24:0)	P	0.570	0.0035
111	PC(17:0_18:2)	P+N	0.441	0.0007
112	PC(17:0_18:3)	P	0.389	0.0002
113	PC(17:1_18:3)	P	0.108	0.0054
114	PC(18:0_16:0)	N	0.404	0.0001
115	PC(18:0_18:1)	N	0.286	0.0003

116	PC(18:0_18:2)	N	0.382	0.0001
117	PC(18:0_18:3)	N	0.440	0.0013
118	PC(18:1_18:2)	N	0.128	0.0002
119	PC(18:2_18:2)	N	0.278	0.0006
120	PC(18:3_18:2)	N	0.237	0.0010
121	PC(18:3_18:3)	N	0.124	0.0008
122	PC(19:0_18:3)	P	0.352	0.0001
123	PC(19:1_16:0)	P	0.326	0.0000
124	PC(19:1_18:1)	P	0.410	0.0002
125	PC(20:0_18:2)	N	0.436	0.0001
126	PC(20:0_18:3)	N	0.429	0.0001
127	PC(21:0e)	P	2.810	0.0003
128	PC(22:0_18:2)	N	0.449	0.0006
129	PC(22:1_12:1)	P	0.577	0.0011
130	PC(23:0e)	P	15.800	0.0005
131	PC(24:0_18:2)	N	1.570	0.0078
132	PC(24:2_12:3)	P	0.217	0.0000
133	PC(26:0_11:4)	P	0.153	0.0033
134	PC(31:1)	P	0.168	0.0013
135	PC(32:0)	P	0.246	0.0060
136	PC(32:1)	P	0.295	0.0000
137	PC(33:1)	P	0.459	0.0003
138	PC(33:2)	P	0.397	0.0005
139	PC(33:3)	P	0.214	0.0056

140	PC(33:4)	P	0.057	0.0024
141	PC(34:0)	P	0.418	0.0029
142	PC(34:1)	P	0.191	0.0049
143	PC(34:3)	P	0.444	0.0001
144	PC(34:5)	P	0.357	0.0025
145	PC(35:5)	P	0.073	0.0057
146	PC(35:6)	P	0.045	0.0041
147	PC(36:0)	P	0.631	0.0000
148	PC(36:2)	P	0.428	0.0007
149	PC(36:3)	P	0.502	0.0009
150	PC(36:4)	P	1.730	0.0041
151	PC(36:6)	P	0.154	0.0034
152	PC(37:0)	P	0.445	0.0001
153	PC(38:2)	P	0.516	0.0061
154	PC(38:3)	P	0.349	0.0006
155	PC(38:4)	P	0.144	0.0024
156	PC(38:5)	P	0.163	0.0039
157	PC(39:2)	P	0.479	0.0011
158	PC(39:3)	P	0.443	0.0004
159	PC(40:2)	P	0.485	0.0046
160	PC(40:3)	P	0.344	0.0009
161	PC(42:1)	P	2.480	0.0078
162	PC(44:2)	P	3.980	0.0031
163	PE(16:0_16:0)	N	0.480	0.0000

164	PE(16:0_18:1)	N	0.215	0.0000
165	PE(16:0_18:2)	P+N	0.428	0.0006
166	PE(16:0_18:3)	P+N	0.361	0.0008
167	PE(17:0_18:2)	N	0.435	0.0004
168	PE(18:0_18:2)	P+N	0.447	0.0009
169	PE(18:0_18:3)	P+N	0.337	0.0003
170	PE(18:1_18:2)	N	0.187	0.0002
171	PE(18:2_18:2)	P+N	16.800	0.0000
172	PE(18:2_21:0)	N	0.453	0.0004
173	PE(18:2_23:0)	P+N	0.621	0.0002
174	PE(18:3_18:2)	P+N	0.182	0.0005
175	PE(18:3_18:3)	P+N	0.115	0.0002
176	PE(20:0_18:2)	N	0.630	0.0052
177	PE(20:0_18:3)	P+N	0.431	0.0003
178	PE(22:0_18:2)	P+N	0.357	0.0011
179	PE(22:0_18:3)	P+N	0.252	0.0003
180	PE(24:0_18:2)	P	1.540	0.0037
181	PE(25:0_18:2)	P+N	2.690	0.0000
182	PE(26:0_18:2)	P+N	3.570	0.0000
183	PE(33:1)	P	0.358	0.0000
184	PE(34:1)	P	0.345	0.0000
185	PE(34:3)	P	2.660	0.0000
186	PE(35:2)	P	0.385	0.0000
187	PE(36:5)	P	0.660	0.0001

188	PE(36:6)	P	0.158	0.0000
189	PE(40:4)	P	2.220	0.0001
190	PE(41:3)	P	0.497	0.0000
191	PE(55:3)	P	10.400	0.0005
192	PG(16:0_16:0)	P+N	1.740	0.0000
193	PG(16:0_17:0)	N	2.410	0.0000
194	PG(16:0_18:1)	N	0.261	0.0004
195	PG(16:0_18:2)	P+N	0.238	0.0002
196	PG(16:0_18:3)	P+N	0.448	0.0008
197	PG(16:0_20:5)	P	0.401	0.0000
198	PG(18:0_16:0)	P	1.600	0.0009
199	PG(18:0_18:2)	N	0.223	0.0002
200	PG(18:2_18:2)	N	0.503	0.0002
201	PG(18:3_18:2)	N	0.388	0.0003
202	PG(34:1)	P	0.289	0.0000
203	PG(36:5)	N	2.290	0.0000
204	PI(14:0_18:2)	N	0.179	0.0001
205	PI(15:0_18:2)	N	0.336	0.0002
206	PI(15:0_18:3)	N	0.203	0.0001
207	PI(16:0_16:0)	P	0.280	0.0001
208	PI(16:0_18:1)	P+N	0.542	0.0047
209	PI(16:0_18:2)	P+N	0.524	0.0005
210	PI(16:0_18:3)	P+N	0.421	0.0001
211	PI(18:0_18:2)	P+N	0.632	0.0013

212	PI(18:0_18:3)	P+N	0.323	0.0015
213	PI(18:1_18:2)	N	0.131	0.0004
214	PI(18:2_18:2)	P	0.113	0.0002
215	PI(18:3_18:2)	P+N	0.084	0.0002
216	PI(18:3_18:3)	P+N	0.150	0.0002
217	PI(18:3_23:0)	N	0.097	0.0000
218	PI(18:4_16:0)	P	2.970	0.0000
219	PI(22:0_18:2)	P+N	0.142	0.0001
220	PI(22:0_18:3)	N	0.083	0.0000
221	PI(24:0_18:2)	P+N	0.333	0.0002
222	PI(24:0_18:3)	N	0.187	0.0001
223	PI(33:2)	P	0.299	0.0001
224	PI(34:1)	P	0.544	0.0004
225	PI(36:3)	P	0.119	0.0000
226	PI(36:6)	P	0.065	0.0000
227	PI(40:3)	P	0.032	0.0000
228	PI(41:2)	P	0.169	0.0000
229	PI(42:2)	P	0.435	0.0002
230	PI(42:3)	P	0.192	0.0000
231	PS(18:2_23:0)	N	0.472	0.0000
232	PS(22:0_18:2)	N	0.245	0.0003
233	PS(22:0_18:3)	N	0.151	0.0042
234	PS(36:3)	N	0.300	0.0000
235	PS(38:6)	N	18.200	0.0000

236	PS(38:8)	N	0.131	0.0001
237	PS(40:3)	P	0.156	0.0000
238	SM(d32:4)	P	2.250	0.0000
239	SPH(d18:2)	P	148.000	0.0000
240	SPH(t18:0)	P	35.300	0.0000
241	SiE(18:3)	P	8.90	0.0005
242	SiE(18:2)	P	8.21	0.0013
243	StE(18:3)	P	4.66	0.002
244	StE(18:2)	P	2.09	0.002
245	TG(12:1e_6:0_18:3)	P	0.572	0.0030
246	TG(14:0_18:2_18:2)	P	0.451	0.0004
247	TG(14:0_18:2_18:3)	P	0.339	0.0001
248	TG(14:0_18:3_18:3)	P	0.310	0.0000
249	TG(15:0_15:0_15:0)	P	1.500	0.0000
250	TG(15:0_16:0_18:3)	P	0.618	0.0023
251	TG(15:0_16:1_18:3)	P	0.653	0.0017
252	TG(15:0_18:2_18:2)	P	0.626	0.0027
253	TG(15:0_18:2_18:3)	P	0.444	0.0003
254	TG(15:0_18:3_18:3)	P	0.329	0.0001
255	TG(16:0_14:0_18:2)	P	0.633	0.0009
256	TG(16:0_14:0_18:3)	P	0.591	0.0001
257	TG(16:0_14:0_22:6)	P	0.174	0.0010
258	TG(16:0_14:2_18:2)	P	15.400	0.0000
259	TG(16:0_16:0_18:1)	P	0.403	0.0020

260	TG(16:0_16:0_18:2)	P	0.484	0.0005
261	TG(16:0_16:0_18:3)	P	0.178	0.0002
262	TG(16:0_17:0_18:1)	P	0.377	0.0001
263	TG(16:0_17:0_18:2)	P	0.427	0.0008
264	TG(16:0_17:1_18:2)	P	0.361	0.0008
265	TG(16:0_18:1_18:1)	P	0.432	0.0004
266	TG(16:0_18:1_18:2)	P	0.247	0.0006
267	TG(16:0_18:1_22:0)	P	0.351	0.0019
268	TG(16:0_18:1_23:0)	P	0.451	0.0013
269	TG(16:0_18:1_24:0)	P	0.496	0.0026
270	TG(16:0_18:2_18:2)	P	0.482	0.0006
271	TG(16:0_18:2_18:3)	P	0.404	0.0002
272	TG(16:0_18:2_21:0)	P	0.508	0.0007
273	TG(16:0_18:2_22:0)	P	0.477	0.0006
274	TG(16:0_18:2_23:0)	P	0.583	0.0015
275	TG(16:0_18:2_24:0)	P	0.654	0.0041
276	TG(16:0_18:3_18:3)	P	0.259	0.0001
277	TG(16:0_18:3_21:0)	P	0.448	0.0001
278	TG(16:0_18:3_22:0)	P	0.415	0.0007
279	TG(16:0_18:3_23:0)	P	0.458	0.0000
280	TG(16:0_6:0_12:1)	P	4.620	0.0000
281	TG(16:0_6:0_12:2)	P	5.190	0.0009
282	TG(16:0_6:0_12:3)	P	0.551	0.0033
283	TG(16:0_8:0_10:2)	P	2.510	0.0054

284	TG(16:1_18:3_18:3)	P	0.564	0.0000
285	TG(17:0_18:2_18:2)	P	0.437	0.0007
286	TG(17:0_6:0_12:2)	P	32.300	0.0001
287	TG(18:0_16:0_18:1)	P	0.341	0.0025
288	TG(18:0_18:0_18:2)	P	0.530	0.0015
289	TG(18:0_18:1_18:2)	P	0.299	0.0005
290	TG(18:0_18:1_24:0)	P	0.518	0.0091
291	TG(18:0_18:2_18:2)	P	0.513	0.0003
292	TG(18:0_18:3_18:3)	P	0.284	0.0001
293	TG(18:0_6:0_12:1)	P	4.590	0.0000
294	TG(18:0_6:0_12:2)	P	0.518	0.0021
295	TG(18:1_18:2_18:2)	P	0.215	0.0003
296	TG(18:1_18:2_22:0)	P	0.537	0.0017
297	TG(18:1_18:2_23:0)	P	0.584	0.0007
298	TG(18:1_18:2_24:0)	P	0.542	0.0089
299	TG(18:2_18:2_21:0)	P	0.447	0.0008
300	TG(18:2_18:2_23:0)	P	0.518	0.0037
301	TG(18:3_11:2_22:2)	P	0.306	0.0000
302	TG(18:3_17:1_18:3)	P	0.399	0.0003
303	TG(18:3_18:2_18:2)	P	0.496	0.0004
304	TG(18:3_18:2_18:3)	P	0.382	0.0001
305	TG(18:3_18:2_20:2)	P	0.237	0.0000
306	TG(18:3_18:2_21:0)	P	0.353	0.0003
307	TG(18:3_18:2_23:0)	P	0.410	0.0002

308	TG(18:3_18:3_18:3)	P	0.325	0.0000
309	TG(18:3_18:3_20:3)	P	0.139	0.0000
310	TG(18:3_18:3_21:0)	P	0.279	0.0000
311	TG(18:3_18:3_22:1)	P	0.189	0.0000
312	TG(18:3_18:3_22:3)	P	0.462	0.0000
313	TG(18:3_18:3_23:0)	P	0.339	0.0000
314	TG(18:4_16:0_16:0)	P	0.324	0.0022
315	TG(18:4_17:1_18:3)	P	0.219	0.0000
316	TG(18:4_6:0_12:0)	P	11.800	0.0000
317	TG(19:0_18:2_18:2)	P	0.590	0.0006
318	TG(19:1_18:3_18:3)	P	0.263	0.0001
319	TG(20:0_16:0_18:1)	P	0.424	0.0018
320	TG(20:0_18:2_18:2)	P	0.525	0.0019
321	TG(20:0_18:2_18:3)	P	0.448	0.0011
322	TG(20:0_18:3_18:3)	P	0.328	0.0001
323	TG(20:1_18:2_18:2)	P	0.363	0.0061
324	TG(22:0_18:2_18:2)	P	0.433	0.0022
325	TG(22:0_18:2_18:3)	P	0.362	0.0002
326	TG(22:0_18:3_18:3)	P	0.250	0.0001
327	TG(24:0_18:2_18:3)	P	0.551	0.0029
328	TG(24:0_18:3_18:3)	P	0.369	0.0000
329	TG(24:1_12:4_12:4)	P	0.007	0.0008
330	TG(24:1_18:3_18:3)	P	0.162	0.0000
331	TG(25:0_16:0_18:1)	P	0.448	0.0010

332	TG(25:0_18:2_18:3)	P	0.631	0.0011
333	TG(25:0_18:3_18:3)	P	0.474	0.0000
334	TG(26:0_18:0_18:3)	P	0.570	0.0029
335	TG(26:0_18:2_18:3)	P	0.658	0.0070
336	TG(26:0_18:3_18:3)	P	0.420	0.0000
337	TG(27:0_18:2_18:3)	P	0.651	0.0030
338	TG(28:0_18:2_18:2)	P	0.548	0.0066
339	TG(28:0_18:2_18:3)	P	0.536	0.0044
340	TG(28:0_18:3_18:3)	P	0.371	0.0000
341	TG(36:2)	P	0.177	0.0013
342	TG(38:3)	P	0.157	0.0002
343	TG(38:5)	P	0.051	0.0011
344	TG(39:6)	P	0.061	0.0000
345	TG(4:0_14:2_22:0)	P	4.310	0.0001
346	TG(4:0_14:2_24:0)	P	8.770	0.0001
347	TG(4:0_16:0_18:2)	P	0.177	0.0012
348	TG(4:0_18:2_18:2)	P	0.062	0.0000
349	TG(6:0_12:1_18:2)	P	4.350	0.0012
350	TG(6:0_12:1_22:0)	P	5.540	0.0000
351	TG(6:0_12:1_24:0)	P	8.030	0.0011
352	TG(6:0_12:2_18:2)	P	3.160	0.0001
353	TG(6:0_12:2_18:3)	P	4.290	0.0008

Table S3 Information of significantly changed lipids in the cotyledon of mung bean seeds between germination day 1 and 4 identified by MALDI-MSI. A paired t-test was conducted to statistically compare six sections from three mung bean seeds in each group by using the paired t test.

No	Name	Detection mode	Fold change (96 h/24 h)	*p* value
1	LPE 18:0	Negative	20.23	0.0003
2	LPE 18:1	Negative	5.59	0.0000
3	LPE 18:2	Negative	15.80	0.0000
4	LPC 16:1	Positive	1.96	0.0000
5	LPC 18:1	Positive	14.65	0.0000
6	LPC 18:2	Positive	31.55	0.0000
7	LPC O-16:1	Positive	1.67	0.0027
8	LPC O-18:2	Positive	2.74	0.0000
9	LPI 13:0	Negative	2.07	0.0026
10	LPI 18:0	Negative	1.78	0.0000
11	LPI 18:1	Negative	1.76	0.0002
12	LPI 18:2	Negative	2.84	0.0000
13	LPI 18:3	Negative	1.88	0.0000
14	LPI 22:1	Negative	1.66	0.0000
15	LPA 16:0	Negative	2.28	0.0000
16	LPA 18:2	Negative	2.67	0.0002
17	LPG 16:0	Negative	1.89	0.0005

18	LPG 22:2	Negative	0.45	0.0025
19	PE 32:2	Negative	0.66	0.0060
20	PE 34:2	Negative	0.51	0.0013
21	PE 34:3	Negative	0.41	0.0045
22	PE 36:2	Negative	0.65	0.0089
23	PE 36:5	Negative	0.64	0.0089
24	PI 24:0	Negative	0.51	0.0043
25	PI 34:2	Negative	0.40	0.0004
26	PI 34:3	Negative	0.26	0.0030
27	PI 34:4	Negative	0.51	0.0001
28	PI 36:2	Negative	0.56	0.0029
29	PI 36:3	Negative	0.33	0.0051
30	PI 36:4	Negative	0.26	0.0029
31	PI 36:5	Negative	0.27	0.0023
32	PI 36:6	Negative	0.50	0.0015
33	PI 40:2	Negative	0.48	0.0051
34	PC 24:0	Positive	0.55	0.0010
35	PC 30:2	Positive	0.64	0.0012
36	PC 34:0	Positive	0.62	0.0996
37	PC 34:1	Positive	0.42	0.0005
38	PC 34:2	Positive	0.47	0.0005
39	PC 34:3	Positive	0.54	0.0005
40	PC 36:2	Positive	0.60	0.0142
41	PC 36:3	Positive	0.44	0.0012

42	PC 36:4	Positive	0.43	0.0016
43	PC 36:5	Positive	0.52	0.0013
44	PC O-41:0	Positive	0.47	0.0003
45	PA 24:0	Negative	1.64	0.0036
46	PA 42:2	Negative	2.56	0.0004
47	PG 34:2	Negative	0.33	0.0009
48	PG 32:0	Negative	1.97	0.0052
49	SM 34:2	Positive	6.46	0.0003
50	DG 34:3	Positive	0.59	0.0008
51	DG 40:9	Positive	0.38	0.0001
52	DG O-32:2	Positive	0.23	0.0001
53	TG 40:1	Positive	3.98	0.0003
54	TG 41:2	Positive	4.04	0.0003
55	TG 42:4	Positive	0.55	0.0000
56	TG 45:5	Positive	0.56	0.0000
57	TG 50:2	Positive	0.30	0.0000
58	TG 50:3	Positive	0.23	0.0001
59	TG 50:4	Positive	0.38	0.0001
60	TG 51:9	Positive	0.62	0.0035
61	TG 52:2	Positive	0.45	0.0012
62	TG 52:3	Positive	0.34	0.0001
63	TG 52:4	Positive	0.33	0.0001
64	TG 52:5	Positive	0.26	0.0000
65	TG 52:6	Positive	0.25	0.0000

66	TG 53:7	Positive	0.60	0.0071
67	TG 53:8	Positive	0.43	0.0003
68	TG 54:3	Positive	0.52	0.0069
69	TG 54:5	Positive	0.43	0.0031
70	TG 54:7	Positive	0.48	0.0080
71	TG 55:10	Positive	0.36	0.0001
72	TG 55:11	Positive	0.43	0.0003
73	TG 55:8	Positive	0.56	0.0075
74	TG 55:9	Positive	0.50	0.0033
75	TG 56:5	Positive	0.50	0.0046

Table S4. Information of significantly changed lipids in the plumule of mung bean seeds between germination day 1 and 4 identified by MALDI-MSI. A paired t-test was conducted to statistically compare six sections from three mung bean seeds in each group.by using the paired t test.

No	Name	Detection mode	Fold change (96 h/24 h)	*p* value
1	LPE 18:0	Negative	2.96	0.0004
2	LPE 18:1	Negative	6.87	0.0002
3	LPE 18:2	Negative	6.40	0.0000
4	LPC 16:1	Positive	1.81	0.0000
5	LPC 18:1	Positive	4.11	0.0000
6	LPC 18:2	Positive	3.13	0.0000
7	LPC O-18:2	Positive	1.65	0.0062
8	LPI 13:0	Negative	1.81	0.0037
9	LPI 18:1	Negative	1.65	0.0002
10	LPI 18:3	Negative	1.93	0.0000
11	LPI 22:1	Negative	2.56	0.0000
12	LPA 16:0	Negative	2.77	0.0001
13	LPA 18:2	Negative	1.94	0.0008
14	LPG 16:0	Negative	2.04	0.0001
15	LPG 22:2	Negative	1.72	0.0000
16	PE 34:2	Negative	0.38	0.0049
17	PE 36:2	Negative	0.28	0.0039

18	PE 36:3	Negative	0.48	0.0091
19	PE 36:4	Negative	0.32	0.0042
20	PE 38:2	Negative	0.57	0.0044
21	PE 43:1	Negative	1.87	0.0014
22	PI 24:0	Negative	0.31	0.0048
23	PI 32:3	Negative	0.55	0.0030
24	PI 34:2	Negative	0.10	0.0015
25	PI 34:3	Negative	0.31	0.0033
26	PI 34:4	Negative	0.57	0.0035
27	PI 36:2	Negative	0.12	0.0020
28	PI 36:3	Negative	0.25	0.0027
29	PI 36:4	Negative	0.27	0.0031
30	PI 36:5	Negative	0.35	0.0022
31	PI 40:2	Negative	0.38	0.0009
32	PC 24:0	Positive	0.58	0.0003
33	PC 34:1	Positive	0.38	0.0053
34	PC 34:2	Positive	0.23	0.0002
35	PC 36:2	Positive	0.28	0.0006
36	PC 36:3	Positive	0.38	0.0097
37	PC 36:4	Positive	0.32	0.0044
38	PC 40:2	Positive	1.79	0.0006
39	PA 24:0	Negative	2.05	0.0030
40	PA 32:0	Negative	3.91	0.0043
41	PA 34:3	Negative	6.83	0.0019

42	PA 36:6	Negative	11.98	0.0015
43	PA 42:2	Negative	2.32	0.0013
44	PG 32:0	Negative	10.81	0.0000
45	PG 34:3	Negative	4.74	0.0000
46	SM 34:2	Positive	0.55	0.0010
47	SM 37:1	Positive	0.65	0.0001
48	DG 39:7	Positive	0.61	0.0078
49	DG 40:9	Positive	0.45	0.0099
50	TG 40:1	Positive	0.52	0.0000
51	TG 42:4	Positive	0.44	0.0000
52	TG 52:2	Positive	0.55	0.0006
53	TG 52:3	Positive	0.36	0.0002
54	TG 52:4	Positive	0.42	0.0022
55	TG 54:4	Positive	0.60	0.0001
56	TG 54:5	Positive	0.63	0.0001
57	TG 54:6	Positive	0.64	0.0001
58	TG 54:8	Positive	1.64	0.0001
